# When Phosphosugars Meet Gold: Synthesis and Catalytic Activities of Phostones and Polyhydroxylated Phosphonite Au(I) Complexes

**DOI:** 10.3390/molecules201219755

**Published:** 2015-11-27

**Authors:** Gaëlle Malik, Angélique Ferry, Xavier Guinchard

**Affiliations:** Institut de Chimie des Substances Naturelles, CNRS UPR 2301, Université Paris-Sud, Université Paris-Saclay, 1 Avenue de la Terrasse, 91198 Gif sur Yvette cedex, France; gaelle.malik@gmail.com (G.M.); angelique.ferry@u-cergy.fr (A.F.)

**Keywords:** gold catalysis, phosphosugars, catalysis, heterocycles, P-stereogeny

## Abstract

The synthesis and characterization of P-chiral phosphonite-, phosphonate- and thiophosphonate-Au(I) complexes are reported. These novel ligands for Au(I) are based on glycomimetic phosphorus scaffolds, obtained from the chiral pool. The catalytic activities of these complexes are shown in the cyclization of allenols and the hydroamination of 2-(2-propynyl)aniline combined with an organocatalyzed reduction to the corresponding 2-phenyl tetrahydroquinoline. All described gold complexes present excellent catalytic activities.

## 1. Introduction

One of the major advances of the 21th century in organic chemistry is undoubtedly the increased importance of gold catalysis. Long believed to be useless for catalysis, gold complexes have emerged as powerful tools for the catalysis of myriads of reactions [1,2,3,4,5,6,7,8,9]. In particular, the gold tolerance towards air, moisture and numerous chemical functionalities renders the use of these catalysts very convenient. However, the bicoordinate linear geometry of gold(I) complexes makes the control of the asymmetry difficult, the chiral ligand being placed in a distal position to the reactive cationic center. Asymmetric approaches have been developed using chiral ligands [10,11,12,13,14], such as chiral phosphines [12,15,16], phosphoramidites [11,17], acyclic diaminocarbenes [18,19,20,21], NHCs [22,23], phospha-helicenes [24,25,26] or phosphate counterions [27,28]. Most of these ligands are based on BINOL, TADDOL and related scaffolds. On the other hand, chiral carbohydrate-type ligands are becoming attractive for asymmetric catalytic purposes, in particular due to their broad availability from the chiral pool, and they have thus been used as ligands in a wide range of catalytic transformations [29,30,31,32,33,34,35,36,37,38]. Surprisingly, to the best of our knowledge, carbohydrates have not been used as ligands for gold, aside from a thioglycoside gold complex developed for biological purposes [39].

**Scheme 1:**
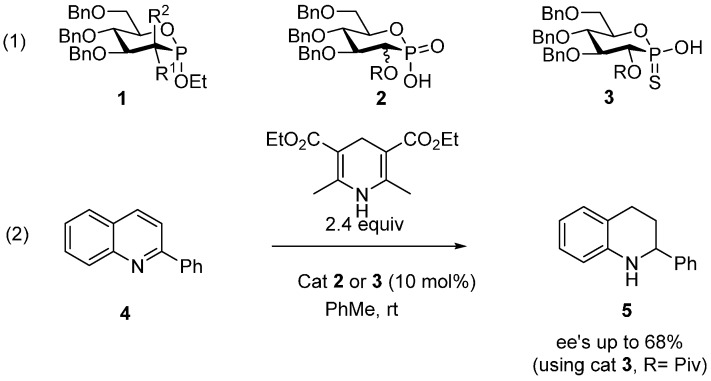
Previously reported results.

In the course of a project aiming at the synthesis of novel glycomimetics [40,41,42,43,44], we recently reported the synthesis of various phosphosugar derivatives bearing anomeric phosphorus functions such as phosphonites **1** and phosphonic acid **2** obtained from the corresponding phosphonate esters (Scheme 1, Equation (1)) [40,41]. More recently, we disclosed the synthesis of configurationally stable carbohydrate-based P-chiral thiophosphonic acids **3** and their use as organocatalysts in the asymmetric reduction of 2-phenylquinoline (**4**) to 2-phenyltetrahydroquinoline (**5**) via asymmetric hydrogen transfer (Scheme 1, Equation (2)) [45,46]. In particular, we could demonstrate the dramatic importance of the configuration of the phosphorus atom on the stereochemical outcome of the reaction.

In this paper, we report the synthesis of novel gold complexes based on the scaffolds **1**–**3** (Scheme 1). Hence, the synthesis of chiral phosphonates, thiophosphonates and phosphonites gold complexes are reported, along with the evidence of their catalytic activities.

## 2. Results and Discussion

**Scheme 2:**
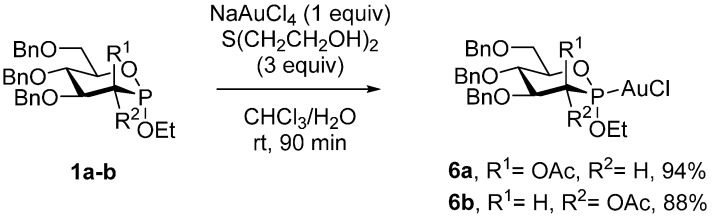
Formation of P-chiral phosphonite Au(I) complexes.

We initiated our study with the synthesis of two novel P-chiral phosphonite gold complexes **6a** and **6b**. For this purpose, the gluco- and manno- phosphonites **1a** and **1b** [41], both bearing an axial (α-configured) P‒OEt bond, were reacted in the presence of sodium tetrachloroaurate and 2,2’-dithioethanol, acting as an internal Au(III) to Au(I) reductant (Scheme 2). The corresponding Au(I) complexes **6a** and **6b** were obtained in 94% and 88% yield, respectively. Only the α-diastereomers were observed, demonstrating the configurational stability of the phosphorus atom. These derivatives appeared fully stable to column chromatography on silica gel and storage, but their oily nature did not allow the determination of X-ray diffraction crystallographic structures. To the best of our knowledge, these structures constitute the first examples of P-chiral phosphonite gold complexes and in a more general manner, one of the rare examples of gold complexes with a phosphonite ligand [39,47,48,49,50,51].

We next turned our attention to the use of P-chiral phosphonite gold complexes **6** in the gold-catalyzed cyclization of allenol **7** leading to tetrahydrofuran **8**. The catalytic activities of these complexes as well as the induction of asymmetry generated by these original ligands were determined. We initiated our study using the manno-configured gold complex **6a**. After activation by AgOTs, the target tetrahydrofuran **8** was obtained in 90% yield and 9% *ee* (Table 1, entry 1). We then screened the effect of the silver salt by using successively AgSbF_6_, AgBF_4_, AgClO_4_ and AgOBz (Table 1, entries 2–5). In most cases, **8** was obtained with good yields, but with moderate enantioselectivities (2%–7% *ee*). The corresponding glucophosphonite gold complex **6b** was next investigated, in order to determine the influence of the C2 configuration on the stereochemical outcome of the reaction and on the catalytic activity. Using the same set of silver salts, the tetrahydrofuran **8** was obtained in good yields (Table 1, entries 6–9), except when silver benzoate was used (Table 1, entry 10), and moderate enantioselectivities (up to 14% *ee*) (Table 1, entry 9). The inversion of configuration at C2 position consequently promoted a drop from 4% *ee* to 14% *ee* using AgClO_4_ as the silver salt. Finally, the effect of the solvent was screened. The use of EtOAc, THF or acetone furnished, in all cases, excellent yields using both catalysts **6a** and **6b** with AgOTs but did not enhance the enantioselectivity (Table 1, entries 11–15).

**Table 1:** Catalytic activity of Au(I) complexes **6a** and **6b** ^1^. 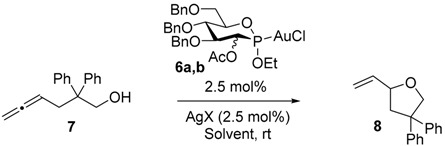

Entry	Cat.	Solvent	AgX	Yield (%) ^2^	*ee* (%)
1	**6a**	PhMe	AgOTs	90	9
2	**6a**	PhMe	AgSbF_6_	62	7
3	**6a**	PhMe	AgBF_4_	94	2
4	**6a**	PhMe	AgClO_4_	68	4
5	**6a**	PhMe	AgOBz	94	5
6	**6b**	PhMe	AgOTs	86	6
7	**6b**	PhMe	AgSbF_6_	64	5
8	**6b**	PhMe	AgBF_4_	70	9
9	**6b**	PhMe	AgClO_4_	66	14
10	**6b**	PhMe	AgOBz	0	-
11	**6a**	EtOAc	AgOTs	98	2
12	**6a**	THF	AgOTs	98	0
13	**6a**	Acetone	AgOTs	94	0
14	**6b**	EtOAc	AgOTs	94	7
15	**6b**	THF	AgOTs	90	4

^1^ Reaction conditions: **6** (0.005 mmol), AgX (0.005 mmol) in PhMe (0.6 mL) were stirred for 10 min, then allenol **7** (0.2 mmol) in PhMe (0.8 mL) was added. ^2^ Isolated yields.

**Scheme 3:**
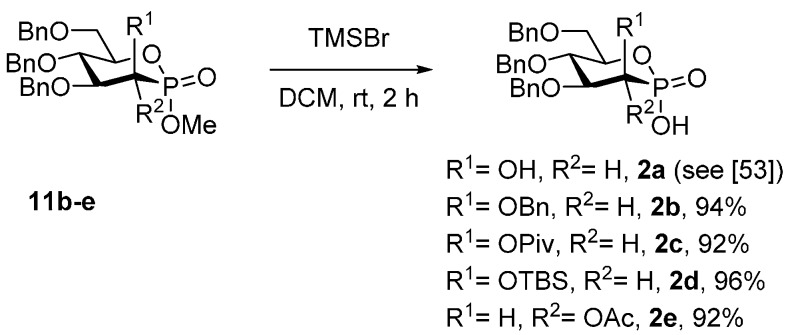
Demethylation of phosphonate esters **11** to phosphonic acids **2.**

In 2007, Toste disclosed an important breakthrough in gold catalysis by using chiral phosphates as gold counterions in asymmetric catalysis [27]. The tight chiral ion pair induced asymmetry in a very efficient manner in cyclizations of allenols. This concept was then used by Echavarren in gold-catalyzed cycloisomerizations. In particular, he could characterize, for the first time, the chiral gold-phosphate complex [52]. Inspired by these precedents, we then envisaged to prepare the phosphonate gold complexes **9** and **10** using both carbohydrate-based phosphonic [45,53] and P-chiral thiophosphonic acids [45]. In this purpose, the mannophosphonate esters **11b**–**d** were deprotected by trimethylsilyl bromide [54,55], furnishing the corresponding acids **2b**–**d** in excellent yields (Scheme 3). By the same way, the acid **2e**, presenting a gluco-like scaffold, was obtained in 92% yield.

The acids **2a**,**b** were then reacted with methyl(triphenylphosphine)gold(I) [56] in dichloromethane, to afford within minutes the two corresponding oily gold complexes **9a**,**b** in quantitative yields. The ^31^P-NMR chemical shifts for the phosphonate functions present a slight shift to lower fields (*i.e.*, 20.2 to 27.7 ppm for **9a**, Table 2, entries 1–2). The chemical shift for the PPh_3_ ligand is around 19 ppm in both cases. Similarly, the reaction of the glucothiophosphonic acid **3a** with Ph_3_PAuMe afforded the related complex **10a** in 98% yield. Interestingly, the ^31^P-NMR chemical shift of the thiophosphonate function shifts dramatically from 79.9 ppm for **3a**, to 51.4 ppm for the gold complex **10a** (Table 2, entries 5–6). This fact is diagnostic for the structural determination of the complex, since such a range of chemical shift is characteristic for the thiolo form, rather than the thiono form [45,57], thereby confirming that the chelating anion is located on the sulfur atom and not on the oxygen. Considering the known strength of the gold-sulfur bond, the covalent nature of the complex is more than likely.

**Table 2:** Formation of Au(I) complexes. 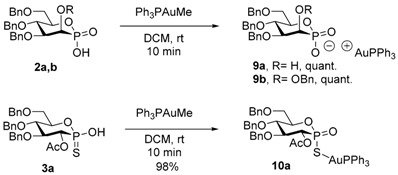

Entry	Compound	^31^P-NMR Chemical Shift
1	**2a**	20.2
2	**9a**	27.7 and 19.2 (PPh_3_)
3	**2b**	20.7
4	**9b**	27.7 and 18.6 (PPh_3_)
5	**3a**	79.9
6	**10a**	51.4 and 37.4 (PPh_3_)

With the confirmation that gold phosphonates are generated upon exposure of the corresponding acids with Ph_3_PAuMe, the catalytic activities of these gold-complexes were evaluated in the tandem gold-catalyzed hydroamination of 2-(2-propynyl)aniline **12**/organocatalyzed reduction of the intermediate dihydroquinoline to tetrahydroquinoline **5**. In this reaction, developed by Gong [58], the chiral phosphoric acid serves both as ligand to the cationic Au(I) complex and as chiral organocatalyst to perform the reduction via hydrogen transfer. In this purpose, a mixture of **12**, catalyst **2**–**3**, Ph_3_PAuMe and Hantzsch ester was stirred in toluene until completion. All phosphonic acids **2b**–**d** furnished the desired tetrahydroquinoline **5** in good yields (Table 3, entries 1–3). The mannophosphonic acid **2b**, protected in the C2 position by a benzyl group, furnished **5** in a low enantiomeric excess of 7% (Table 3, entry 1). The *ee* increased to 14% when the bulky Piv-protected acid **2c** was used (Table 3, entry 2) but decreased to 4% in the case of the TBS-protected acid **2d** (Table 3, entry 3). For comparison with the manno-series, the glucophosphonic acid **2e** was evaluated and furnished the product in an excellent 93% yield and with 5% enantiomeric excess (Table 3, entry 4).

We then examined the use of P-stereogenic thiophosphonic acids **3a** and **3b**, both presenting an equatorial acidic OH function and an axial P = S double bond. When the reaction was performed at room temperature using **3a** or **3b**, no conversion of **12** to **5** was observed (Table 3, entry 5). The increase of the temperature to 60 °C furnished only trace amounts of the heterocycle **5** (Table 3, entry 6). Echavarren previously reported the catalytic inactivity of gold phosphates in cycloisomerization reactions involving alkynes [52]. He showed that the addition of methanol to the reaction restores the catalytic activity of the gold phosphates in these reactions, presumably by facilitating the ligand exchange through activation via H-bonding of the phosphate. In the complex **3b**, the thiophosphonate-Au(I) complex is covalently bounded by its sulfur atom (*vide supra*). The gold-sulfur bond has been the subject of numerous studies [59], and is usually considered as strong. It is consequently likely that the ligand exchange does not occur, thereby inhibiting the gold-catalyzed hydroamination step. We then hypothesized that addition of methanol to the reaction mixture would potentially restore the catalytic activity. A moderate 25% yield was indeed obtained when the reaction was performed in a 1/1 toluene/methanol mixture, with a low 6% enantiomeric excess (Table 3, entry 7). When methanol was used as the sole solvent using catalysts **3a** and **3b**, yields typically ranging to 27%–59% were obtained (Table 3, entries 8–9). However, as expected, the enantiomeric excess dropped to 0% in both cases. Indeed, this class of reaction is known to proceed typically in aprotic solvents, in order to enable chiral H-bonded supramolecular assembly between the substrate, the acid and the Hantzsch ester [46]. It seems consequently unlikely good enantiomeric excesses can be reached using our thiophosphonic acids as organocatalysts. However, these results testify to the excellent catalytic activities of the gold-phosphonates **9** in the first step of this tandem sequence.

**Table 3:** Catalytic activity of phosphonate-Au(I) complexes ^1^. 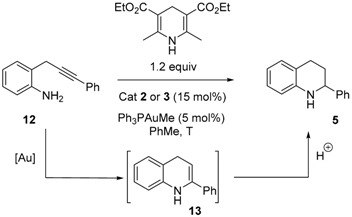

Entry	Cat.	T (°C)	Yield (%) ^2^	*ee* (%)
1	**2b**	rt	99	7
2	**2c**	rt	99	14
3	**2d**	rt	80	4
4	**2e**	rt	93	15
5	**3a** or **3b**	rt	0	-
6	**3a** or **3b**	60	traces	-
7 ^3^	**3a**	60	25%	6
8 ^4^	**3a**	60	27%	0
9 ^4^	**3b**	60	59%	0
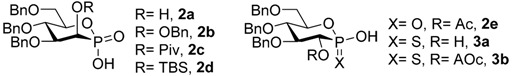				

^1^ Reaction conditions: Phosphonic acid **2** (0.15 equiv.) in PhMe (0.6 mL), Ph_3_PAuMe (0.05 equiv.), Hantzsch ester (1.2 equiv.) and aniline **12** (1 equiv.). ^2^ Isolated yields. ^3^ Reaction performed in a 1/1 mixture of PhMe/MeOH. ^4^ Reaction performed in MeOH.

## 3. Experimental Section

### 3.1. General Information

Reactions were performed using oven dried glassware under an atmosphere of argon. All separations were carried out under flash-chromatographic conditions on silica gel (prepacked column, 230–400 mesh, Interchim, Montluçon, France) at medium pressure (20 psi) with use of a CombiFlash Companion (Serlabo, Entraigues, France). Reactions were monitored by thin-layer chromatography on silica gel plates which were rendered visible by ultraviolet and spraying with vanillin (15%) + sulfuric acid (2.5%) in EtOH followed by heating. THF, CH_2_Cl_2_, DMF, MeOH and MTBE (*i.e.*, methyl *tert*-butyl ether) were purchased from Fisher Scientific (Ilkirch, France) at the highest commercial quality and used without further purification. Reagent-grade chemicals were obtained from diverse commercial suppliers (mainly Sigma-Aldrich, Saint-Quentin-Fallavier, France) and were used as received. ^1^H-NMR and ^13^C-NMR spectra were recorded at 300/500 and 125/75 MHz, respectively, at 298 K unless otherwise stated. Chemical shifts are given in ppm (δ) and are referenced to the internal solvent signal or to TMS used as an internal standard. Multiplicities are declared as follow: s (singlet), brs (broad singlet), d (doublet), t (triplet), q (quadruplet), dd (doublet of doublet), ddd (doublet of doublet of doublet), dt (doublet of triplet), m (multiplet), AB = AB quartet, AB*X* = ABX system. Coupling constants *J* are given in Hz. Carbon multiplicities were determined by DEPT135 experiment. Diagnostic correlations were obtained by two-dimensional COSY, HSQC and NOESY experiments. Infrared spectra (IR) were recorded on a Perkin-Elmer FT-IR system (Villebon-sur-Yvette, France) using a diamond window Dura SamplIR II and the data are reported in reciprocal centimeters (cm^−1^). Optical rotations were measured on a MCP 300 polarimeter (Anton Paar, Courtaboeuf, France) at 589 nm. [α]_D_ is expressed in deg·cm^3^·g^−1^·dm^−1^ and *c* is expressed in g/100 cm^3^. High resolution mass spectra (HRMS) were recorded using a Micromass LCT Premier XE instrument (Waters, Guyancourt, France) and were determined by electrospray ionization (ESI).

### 3.2. Synthesis of Chloro[(2S,3R,4S,5S,6R)-4,5-bis(benzyloxy)-6-((benzyloxy)methyl)-2-ethoxy-1,2-oxaphosphinan-3-yl acetate] gold *(**6a**)*

To a solution of NaAuCl_4_ (71 mg, 0.178 mmol) in dry H_2_O (8 mL) was added slowly (15 min) at 0 °C thiodiglycol (65 mg, 0.54 mmol) and the resulting solution was stirred for 1 h. A solution of phosphonite **1a** (96 mg, 0.178 mmol) in CHCl_3_ (3 mL) was then added and the resulting solution was further stirred at rt for 90 min. The phases were separated and the aqueous layer was extracted by CHCl_3_. Combined organic layers were dried over MgSO_4_, filtered and evaporated, yielding the product **6a** as a colourless oil (130 mg, 0.167 mmol, 94%). ${\left[\alpha \right]}_{\text{D}}^{24}$ = −3.0 (*c* 0.95, CHCl_3_); IR (neat) ν_max_ 3031, 2931, 2868, 1752, 1367, 1212, 1093, 1019, 961 cm^−1^; ^1^H-NMR (300 MHz, CDCl_3_) δ 7.36–7.26 (m, 13H), 7.17–7.10 (m, 2H), 5.68 (t, *J =* 2.9 Hz, 1H), 4.82 (d, *J =* 10.5 Hz, 1H), 4.72 (d, *J =* 10.9 Hz, 1H), 4.63 (d, *J =* 12.1 Hz, 1H), 4.55 (d, *J =* 10.8 Hz, 1H), 4.51 (d, *J =* 11.0 Hz, 1H), 4.49 (d, *J =* 10.5 Hz, 1H), 4.34–4.16 (m, 3H), 4.04 (dd, *J =* 3.1 and 9.4 Hz, 1H), 3.94 (t, *J* = 9.6 Hz, 1H), 3.83 (ddd, *J =* 1.1, 4.1 and 11.3 Hz, 1H), 3.71 (dd, *J =* 1.8 and 11.2 Hz, 1H), 2.24 (s, 3H), 1.26 (t, *J =* 7.1 Hz, 3H). ^13^C-NMR (125 MHz, CDCl_3_) δ 159.6 (d, *J =* 4.9 Hz, Cq), 137.7 (Cq), 137.6 (Cq), 137.0 (Cq), 128.7 (CH), 128.6 (CH), 128.4 (CH), 128.3 (CH), 128.2 (CH), 128.0 (CH), 128.0 (CH), 78.8 (d, *J =* 11 Hz, CH), 78.5 (CH), 76.0 (CH_2_), 73.8 (CH_2_), 73.4 (d, *J =* 3.8 Hz, CH), 72.4 (CH_2_), 69.0 (d, *J* = 7.7 H, CH_2_), 68.8 (CH_2_), 68.5 (d, *J =* 69.2 Hz, CH), 21.0 (CH_3_), 16.6 (d, *J =* 6.0 Hz, CH_3_). ^31^P-NMR (122 MHz, CDCl_3_) δ 131.9; HRMS (ESI-TOF) calcd for C_30_H_39_AuClNO_7_P 788.1818 [M + NH_4_]^+^, found 788.1826.

### 3.3 Synthesis of Chloro[(2S,3S,4S,5S,6R)-4,5-bis(benzyloxy)-6-((benzyloxy)methyl)-2-ethoxy-1,2-oxaphosphinan-3-yl acetate] gold *(**6b**)*

To a solution of NaAuCl_4_ (52 mg, 0.13 mmol) in dry H_2_O (6 mL) was added slowly (15 min) at 0 °C thiodiglycol (48 mg, 0.39 mmol) and the resulting solution was stirred for 1 h. A solution of phosphonite **1b** (70 mg, 0.13 mmol) in CHCl_3_ (2 mL) was then added and the resulting solution was further stirred at rt for 90 min. The phases were separated and the aqueous layer was extracted by CHCl_3_. Combined organic layers were dried over MgSO_4_, filtered and evaporated, yielding the product **6b** as a colourless oil (88 mg, 0.114 mmol, 88%). ${\left[\alpha \right]}_{\text{D}}^{24}$ = +65.7 (*c* 0.01, CHCl_3_); IR (neat) ν_max_ 2924, 2854, 1755, 1215, 1048 cm^−1^; ^1^H-NMR (300 MHz, CDCl_3_) δ 7.37–7.26 (m, 11H), 7.26–7.20 (m, 2H), 7.15–7.08 (m, 2H), 5.39 (d, *J =* 10.3 Hz, 1H), 4.83 (d, *J =* 11.0 Hz, 1H), 4.80 (d, *J =* 10.2 Hz, 1H), 4.68 (d, *J =* 11.3 Hz, 1H), 4.64–4.50 (m, 3H), 4.40–4.25 (m, 2H), 4.25–4.16 (m, 1H), 4.03 (dt, *J =* 2.8 and 9.4 Hz, 1H), 3.88 (t, *J =* 9.7 Hz, 1H), 3.86–3.79 (m, 1H), 3.71 (dd, *J =* 1.8 and 11.2 Hz, 1H), 2.01 (s, 3H), 1.40 (t, *J =* 7.1 Hz, 3H). ^13^C-NMR (125 MHz, CDCl_3_) δ 169.0 (d, *J =* 3.3 Hz, Cq), 137.8 (Cq), 137.5 (Cq), 137.4 (Cq), 128.8 (CH), 128.7 (CH), 128.3 (CH), 128.2 (CH), 128.1 (CH), 128.1 (CH), 127.7 (CH), 80.9 (CH), 77.7 (d, *J =* 11.0 Hz, CH), 77.4 (CH), 76.3 (CH_2_), 76.1 (CH_2_), 74.0 (CH_2_), 72.6 (d, *J =* 61.5 Hz, CH), 69.4 (CH_2_), 68.5 (d, *J =* 8.8 Hz, CH_2_), 20.7 (CH_3_), 16.8 (d, *J =* 5.5 Hz, CH_3_). ^31^P-NMR (122 MHz, CDCl_3_) δ 129.5; HRMS (ESI-TOF) calcd for C_30_H_35_AuClO_7_P 776.2051 [M − Cl + MeCN]^+^, found 776.2079.

### 3.4. Representative Procedure for Gold Catalyzed-Cyclization of Allenol ***7***

To a solution of chlorophosphonite-gold complex **6a** (3.8 mg, 0.005 mmol) in PhMe (0.6 mL) at rt was added AgOTs (1.4 mg, 0.005 mmol) and the resulting solution was stirred for 10 min. The allenol **7** (50 mg, 0.2 mmol) in PhMe (0.8 mL) was then added and the solution was stirred for 1 h until TLC indicated full conversion. It was then directly purified on silica gel (eluent: EtOAc/heptane, 1/8), providing **8** (45 mg, 0.18 mmol, 90%) as a colorless oil, which data were in full agreement with the literature [60]. The enantiomeric excess was determined to be 9% by chiral HPLC (Chiralcel AD-H, eluent heptane/*i*-PrOH, 99.5/0.5).

### 3.5. Synthesis of Phosphonic Acids ***2***

To a solution of **11** in CH_2_Cl_2_, was added bromotrimethylsilane. The resulting mixture was stirred for the indicating time at rt. The reaction was then quenched with water. The two layers were separated and the aqueous layer was reextracted two times with EtOAc. Organic layer was washed with HCl 2 N and then dried over MgSO_4_, filtered and concentrated, affording pure acid **2**.

*(2S,3R,4S,5S,6R)-3,4,5-Tris(benzyloxy)-6-((benzyloxy)methyl)-2-hydroxy-1,2-oxaphosphinane 2-oxide* (**2b**). **2b** was done according to the general procedure by reaction of **11b** (200 mg, 0.34 mmol), CH_2_Cl_2_ (5 mL) and bromotrimethylsilane (260 mg, 1.7 mmol, 5 equiv.). The resulting mixture was stirred 2 h. **2b** was obtained as a colorless oil (184 mg, 0.32 mmol, 94%). ${\left[\alpha \right]}_{\text{D}}^{22}$ = −18.3° (*c* 2.2, CHCl_3_); IR (neat) ν_max_ 3032, 2869, 1497, 1454, 1363, 1208, 1096, 997, 908, 735, 696 cm^−1^; ^1^H-NMR (300 MHz, CDCl_3_) 10.7 (bs, 1H), 7.52–7.42 (m, 2H), 7.40–7.23 (m, 16H), 7.23–7.11 (m, 2H), 5.00 (d, *J* = 12.1 Hz,1H), 4.91 (d, *J* = 10.7 Hz, 1H), 4.80 (d, *J* = 12.0 Hz, 1H), 4.68 (d, *J* = 12.1 Hz, 1H), 4.61–4.48 (m, 4H), 4.30–4.14 (m, 3H), 4.09–3.98 (m, 1H), 3.92–3.82 (m, 1H), 3.77 (d, *J* = 11.3 Hz, 1H); ^13^C-NMR (75 MHz, CDCl_3_) 138.2 (C_q_), 138.2 (C_q_), 138.0 (C_q_), 137.6 (C_q_), 128.5 (5 × CH), 128.5 (6 × CH), 128.1 (2 × CH), 128.0 (2 × CH), 128.0 (CH), 127.9 (CH), 127.8 (3 × CH), 81.8 (d, *J* = 9.3 Hz, CH), 77.4 (d, *J* = 3.8 Hz, CH), 75.5 (CH_2_), 74.3 (CH_2_), 74.2 (CH), 73.7 (CH_2_), 72.3 (CH_2_), 70.9 (d, *J* = 145.5 Hz, CH,), 69.3 (d, *J* = 9.3 Hz, CH_2_); ^31^P-CPD-NMR (122 MHz, CDCl_3_) 20.7 ppm; HRMS (ESI-TOF) calcd for C_33_H_34_O_7_P [M − H]^−^ 574.2120, found 574.2126.

*(2S,3R,4S,5S,6R)-4,5-Bis(benzyloxy)-6-((benzyloxy)methyl)-2-hydroxy-2-oxido-1,2-oxaphosphinan-3-yl pivalate* (**2c**). **2c** was done according to the general procedure by reaction of **11c** (39.9 mg, 0.069 mmol), CH_2_Cl_2_ (1 mL) and bromotrimethylsilane (45 µL, 0.35 mmol, 5 equiv.). The resulting mixture was stirred 2 h. **2c** was obtained as a colorless oil (36.1 mg, 0.063 mmol, 92%). *R_f_* = 0.18 (EtOAc); ${\left[\alpha \right]}_{\text{D}}^{20}$ = +0.4° (*c* 1.3, CHCl_3_); IR (neat) ν_max_ 3031, 2973, 2871, 1739, 1452, 1277, 1208, 1145, 1097, 1005, 736, 697 cm^−1^; ^1^H-NMR (300 MHz, CDCl_3_) 7.35–7.24 (m, 13H), 7.16–7.13 (m, 2H), 5.88 (dd, *J* = 11.1 and 1.7 Hz, 1H), 4.82 (d, *J* = 10.7 Hz, 1H), 4.74 (d, *J* = 10.9 Hz, 1H), 4.66 (d, *J* = 12.1 Hz, 1H), 4.51 (d, *J* = 12.4 Hz, 1H), 4.51 (d, *J* = 10.4 Hz, 1H), 4.49 (d, *J* = 10.9 Hz, 1H), 4.23–4.20 (m, 1H), 4.12–4.02 (d, 2H), 3.90–3.84 (m, 1H), 3.75 (d, *J* = 10.9 Hz, 1H), 1.23 (s, 9H); ^13^C-NMR (75 MHz, CDCl_3_) 177.2 (d, *J* = 4.4 Hz, C_q_), 138.3 (C_q_), 138.1 (C_q_), 137.8 (C_q_), 128.5 (4 × CH), 128.5 (2 × CH), 128.3 (4 × CH), 128.0 (CH), 127.9 (CH), 127.8 (CH), 127.7 (2x CH), 80.3 (d, *J* = 7.1 Hz, CH), 77.1 (d, *J* = 3.8 Hz, CH), 75.7 (CH_2_), 73.9 (CH), 73.6 (CH_2_), 71.7 (CH_2_), 69.1 (d, *J* = 9.3 Hz, CH_2_), 63.7 (d, *J* = 148.8 Hz, CH), 39.3 (C_q_), 27.3 (CH_3_); ^31^P-CPD-NMR (122 MHz, CDCl_3_) 17.1 ppm; HRMS (ESI-TOF) calcd for C_31_H_36_O_8_P [M − H]^−^ 567.2153, found 567.2126.

*(2S,3R,4S,5S,6R)-4,5-Bis(benzyloxy)-6-((benzyloxy)methyl)-3-((tert-butyldimethylsilyl)oxy)-2-hydroxy-1,2-oxaphosphinane 2-oxide* (**2d**). **2d** was done according to the general procedure by reaction of **11d** (32 mg, 0.052 mmol), CH_2_Cl_2_ (1 mL) and bromotrimethylsilane (40 mg, 0.26 mmol, 5 equiv.). The resulting mixture was stirred 2 h. **2d** was obtained as a colorless oil (30 mg, 0.05 mmol, 96%). ${\left[\alpha \right]}_{\text{D}}^{22}$ = +34.9° (*c* 1.1, CHCl_3_); IR (neat) ν_max_ 3065, 3032, 2928, 2884, 2856, 1497, 1454, 1362, 1250, 1208, 1138, 1097, 997, 833, 781, 734, 697 cm^−1^; ^1^H-NMR (500 MHz, CDCl_3_) 7.86–7.64 (bs, 1H), 7.31–7.14 (m, 13H), 7.12–7.04 (m, 2H), 4.77 (d, *J* = 11.0 Hz, 1H), 4.66 (d, *J* = 11.6 Hz, 1H), 4.58 (d, *J* = 12.2 Hz, 1H), 4.57 (d, *J* = 11.3 Hz, 1H), 4.47 (d, *J* = 11.0 Hz, 1H), 4.44 (d, *J* = 12.2 Hz, 1H), 4.29 (dd, *J* = 9.2 and 1.5 Hz, 1H), 4.13 (t, *J* = 9.2 Hz, 1H), 4.11–4.06 (m, 1H), 3.89 (dd, *J* = 9.2 and 1.5 Hz, 1H), 3.77 (td, *J* = 11.3 and 3.1 Hz, 1H), 3.66 (d, *J* = 11.0 Hz, 1H), 0.85 (s, 9H), 0.07 (s, 3H), 0.04 (s, 3H); ^13^C-NMR (75 MHz, CDCl_3_) 138.5 (C_q_), 138.2 (C_q_), 138.0 (C_q_), 128.5 (4 × CH), 128.5 (3 × CH), 128.2 (CH), 127.9 (3 × CH), 127.8 (CH), 127.7 (2 × CH), 127.6 (CH), 82.2 (d, *J* = 9.3 Hz, CH), 77.4 (d, *J* = 3.3 Hz, CH), 75.5 (CH_2_), 73.9 (CH), 73.5 (CH_2_), 72.9 (CH_2_), 69.3 (d, *J* = 9.9 Hz, CH_2_), 66.6 (d, *J* = 150.4 Hz, CH), 25.9 (3 × CH_3_), 18.5 (C_q_), −4.7 (CH_3_); ^31^P-CPD-NMR (122 MHz, CDCl_3_) 21.8 ppm.

*(2S,3S,4S,5S,6R)-4,5-Bis(benzyloxy)-6-((benzyloxy)methyl)-2-hydroxy-2-oxido-1,2-oxaphosphinan-3-yl acetate* (**2e**). **2e** was done according to the general procedure by reaction of **11e** (38 mg, 0.07 mmol), CH_2_Cl_2_ (1 mL) and bromotrimethylsilane (54 mg, 0.35 mmol, 5 equiv.). The resulting mixture was stirred 2 h. **2e** was obtained as a colorless oil (34 mg, 0.065 mmol, 92%). ${\left[\alpha \right]}_{\text{D}}^{22}$ = +51.9° (*c* 0.7, CHCl_3_); IR (neat) ν_max_ 2924, 1754, 1497, 1454, 1367, 1225, 1108, 1050, 1000, 889, 845, 742, 698 cm^−1^; ^1^H-NMR (500 MHz, CDCl_3_) 7.30–7.11 (m, 13H), 7.11–7.01 (m, 2H), 5.33 (t, *J* = 10.1Hz,1H), 4.75 (d, *J* = 11.6 Hz, 1H), 4.73 (d, *J* = 10.7 Hz, 1H), 4.60 (d, *J* = 11.3 Hz, 1H), 4.52 (d, *J* = 12.5 Hz, 1H), 4.49 (d, *J* = 11.0 Hz, 1H), 4.42 (d, *J* = 11.9 Hz, 1H), 4.18–4.11 (m, 1H), 3.92 (t, *J* = 9.8 Hz, 1H), 3.83 (t, *J* = 9.8 Hz, 1H), 3.74 (d, *J* = 11.0 Hz, 1H), 3.64 (d, *J* = 10.7 Hz, 1H), 1.92 (s, 3H); ^13^C-NMR (75 MHz, MeOD) 169.8 (C_q_), 138.1 (C_q_), 137.7 (2 × C_q_), 128.7 (4 × CH), 128.6 (2 × CH), 128.6 (2 × CH), 128.5 (2 × CH), 128.4 (CH), 128.2 (CH), 128.0 (2 × CH), 128.0 (CH), 81.8 (d, *J* = 9.3 Hz, CH), 78.0 (CH), 76.5 (CH), 76.2 (CH_2_), 75.7 (CH_2_), 73.7 (CH_2_), 68.5 (d, *J* = 148.2 Hz, CH), 68.5 (d, *J* = 9.3 Hz, CH_2_), 20.7 (CH_3_); ^31^P-CPD-NMR (122 MHz, CDCl_3_) 16.8 ppm; HRMS (ESI-TOF) calcd for C_28_H_30_O_8_P [M − H]^−^ 525.1678, found 525.1662.

### 3.6. Formation of Gold Complexes ***9*** and ***10***

*(((2S,3S,4S,5S)-4,5-Bis(benzyloxy)-6-((benzyloxy)methyl)-3-hydroxy-2-oxido-1,2-oxaphosphinan-2-yl)oxy)(triphenyl phosphanyl)gold* (**9a**). To a solution of acid **2a** (25.2 mg, 0.052 mmol) in DCM (4 mL) was added Ph_3_PAuMe (24.7 mg, 0.052 mmol) and the resulting mixture was stirred at rt for 20 min, then evaporated under vacuum. **9a** was then obtained (49 mg, 0.052 mmol, quant.) as an oil. ^1^H-NMR (500 MHz, CDCl_3_) 7.60–7.39 (m, 15H), 7.39–7.15 (m, 9H), 4.91 (d, *J* = 10.7 Hz, 1H), 4.76 (d, *J* = 11.6 Hz, 1H), 4.72–4.62 (m, 2H), 4.58 (d, *J* = 10.7 Hz, 1H), 4.52 (d, *J* = 12.2 Hz, 1H), 4.40 (d, *J* = 8.2 Hz, 1H), 4.30–4.20 (m, 1H), 4.19–4.07 (m, 2H), 3.87 (d, *J* = 10.2 Hz, 1H), 3.76 (d, *J* = 10.7 Hz, 1H); ^13^C-NMR (75 MHz, CDCl_3_) 138.8 (C_q_), 138.7 (C_q_), 138.4 (C_q_), 134.3 (d, *J* = 13.2 Hz, CH), 132.3 (CH), 129.5 (d, *J* = 12.1 Hz, CH), 128.5 (CH), 128.4 (CH), 128.4 (CH), 128.1 (CH), 128.0 (CH), 127.9 (CH), 127.8 (CH), 127.6 (CH), 127.5 (CH), 82.8 (d, *J* = 4.1 Hz, CH), 75.7 (d, *J* = 3.8 Hz, CH), 75.4 (CH_2_), 74.7 (CH), 75.4 (CH_2_), 71.7 (CH_2_), 70.0 (d, *J* = 9.3 Hz, CH_2_), 65.4 (d, *J* = 141.1 Hz, CH); ^31^P-CPD-NMR (122 MHz, CDCl_3_) 27.8, 19.2 ppm.

*(Triphenylphosphanyl)(((2S,3S,4S,5S)-3,4,5-tris(benzyloxy)-6-((benzyloxy)methyl)-2-oxido-1,2-oxaphosphinan-2-yl)oxy)gold* (**9b**). To a solution of acid **2b** (18 mg, 0.031 mmol) in DCM (3 mL) was added Ph_3_PAuMe (14.9 mg, 0.031 mmol) and the resulting mixture was stirred at rt for 20 min, then evaporated under vacuum. **9b** was then obtained (32.8 mg, 0.031 mmol, quant.) as an oil. ^1^H-NMR (500 MHz, CDCl_3_) 7.60–7.39 (m, 15H), 7.39–7.05 (m, 20H), 5.04 (d, *J* = 12.2 Hz, 1H), 4.92 (d, *J* = 10.7 Hz, 1H), 4.87 (d, *J* = 12.2 Hz, 1H), 4.65 (d, *J* = 11.9 Hz, 1H), 4.55 (d, *J* = 10.7 Hz, 1H), 4.53–4.44 (m, 3H), 4.30–4.20 (m, 1H), 4.22–4.10 (m, 3H), 3.87 (d, *J* = 9.2 Hz, 1H), 3.76 (d, *J* = 11.0 Hz, 1H); ^13^C-NMR (75 MHz, CDCl_3_) 138.8 (3C_q_), 134.4 (d, *J* = 13.2 Hz, CH), 132.3 (CH), 129.5 (d, *J* = 12.6 Hz, CH), 128.5 (CH), 128.4 (CH), 128.3 (CH), 128.1 (CH), 128.0 (CH), 127.9 (CH), 127.6 (CH), 127.5 (CH), 127.5 (CH), 83.2 (d, *J* = 8.8 Hz, CH), 76.3 (CH), 75.3 (CH_2_), 75.2 (CH), 73.6 (CH_2_), 71.9 (d, *J* = 141.6 Hz, CH), 71.8 (CH_2_), 70.2 (d, *J* = 10.4 Hz, CH); ^31^P-NMR (122 MHz, CDCl_3_) δ 27.7; 18.6.

*(((2S,3R,4S,5S)-3-Acetoxy-4,5-bis(benzyloxy)-6-((benzyloxy)methyl)-2-oxido-1,2-oxaphosphinan-2-yl)thio)(triphenyl-l5-phosphanyl)gold* (**10a**). To a solution of thioacid **3a** (32 mg, 0.059 mmol) in DCM (4 mL) was added Ph_3_PAuMe (28.0 mg, 0.059 mmol) and the resulting mixture was stirred at rt for 20 min, then evaporated under vacuum. **10a** was then obtained (58 mg, 0.058 mmol, 98%) as an oil. ^1^H-NMR (300 MHz, CDCl_3_) 7.62–7.39 (m, 15H), 7.37–7.21 (m, 13H), 7.20–7.11 (m, 2H), 5.55 (t, *J* = 10.7 Hz,1H), 4.83 (d, *J* = 11.3 Hz, 1H), 4.82 (d, *J* = 10.5 Hz, 1H), 4.62 (d, *J* = 12.1 Hz, 1H), 4.62 (d, *J* = 10.5 Hz, 1H), 4.49 (d, *J* = 12.1 Hz, 1H), 4.42–4.32 (m, 1H), 4.07 (td, *J* = 3.6 and 9.4 Hz, 1H), 3.96 (t, *J* = 9.6 Hz, 1H), 3.89 (dt, *J* = 3.2 and 11.1 Hz, 1H), 3.73 (dd, *J* = 1.7 and 11.3 Hz, 1H), 1.80 (s, 3H); ^13^C-NMR (75 MHz, MeOD) 169.4 (d, *J* = 4.4 Hz C_q_), 138.4 (C_q_), 138.3 (C_q_), 138.1 (C_q_), 134.4 (d, *J* = 14.3 Hz, CH), 132.1 (CH), 129.5 (d, *J* = 12.1 Hz, CH), 128.6 (CH), 128.5 (CH), 128.1 (CH), 128.0 (CH), 127.8 (CH), 127.7 (CH), 83.7 (d, *J* = 11.5 Hz, CH), 78.5 (CH), 76.8 (d, *J* = 6.0 Hz, CH), 76.1 (CH_2_), 75.6 (CH_2_), 73.8 (d, *J* = 104.3 Hz, CH), 73.6 (CH_2_), 69.1 (d, *J* = 11.5 Hz, CH_2_), 20.9 (CH_3_); ^31^P-CPD-NMR (122 MHz, CDCl_3_) 51.4, 37.4 ppm.

The supplementary material contains all NMR spectra.

### 3.7. Representative Procedure for Gold/Acid Catalyzed-Cyclization of ***12*** in ***5***

To a solution of phosphonic acid **2** (0.15 equiv) in PhMe (0.6 mL) at rt were added successively Ph_3_PAuMe (0.05 equiv.), hantzsch ester (1.2 equiv.) and aniline **12** (1 equiv.). The resulting solution was stirred overnight and then concentrated under vaccum. It was then directly purified on silica gel (eluent: EtOAc/heptane, 5/95), providing **5** as a colorless oil, which data were in full agreement with the literature [47]. The enantiomeric excess was determined by chiral HPLC (Chiralpack IB, eluent heptane/*i*-PrOH, 95/5). 

## 4. Conclusions

In this paper, we have described the synthesis of novel Au(I) complexes based on chiral phosphosugar scaffolds and reported preliminar activities in catalysis. P-Chiral phosphonite gold complexes **6** proved very active in the cyclization of allenol to tetrahydrofuran, but furnished low enantioselectivities. Phosphonate and thiophosphonate gold complexes were also reported and characterized by ^1^H- and ^31^P-NMR. They were then generated *in situ* and their catalytic activities were demonstrated in the tandem gold-catalyzed hydroamination/organocatalyzed reduction by hydrogen transfer sequence of 2-(2-propynyl)aniline **12** to tetrahydroisoquinoline **5**. While the use of phosphonic acids **2** afforded the product in excellent yields, the use of thiophosphonic acid **3a** resulted in a loss of the catalytic activity due to the strength of the sulfur-gold bond. Though moderate enantioselectivities were obtained using these phosphosugar derivatives as Au(I) ligands, this report is the first use of carbohydrates mimics as ligands for gold catalysis and the synthesized gold complexes bearing these ligands showed excellent catalytic activities. These results may pave the way to the discovery of innovative phosphorus chiral ligands obtained from the chiral pool for catalysis.

## Supplementary Materials

Supplementary materials can be accessed at: http://www.mdpi.com/1420-3049/20/12/19755/s1.
