# Supplementary material for: When Phosphosugars Meet Gold: Synthesis and Catalytic Activities of Phostones and Polyhydroxylated Phosphonite Au(I) Complexes
Source: Molecules. 2015 Nov 27;20(12):21082–93. doi: 10.3390/molecules201219755 (PMC6332187; doi:10.3390/molecules201219755)
Supplement: Supplementary file 1 [file molecules-20-19755-s001.pdf]

Supplementary Materials: When Phosphosugars Meet Gold: Synthesis and Catalytic Activities of Phostones and Polyhydroxylated Phosphonite Au(I) Complexes

Gaëlle Malik, Angélique Ferry and Xavier Guinchard

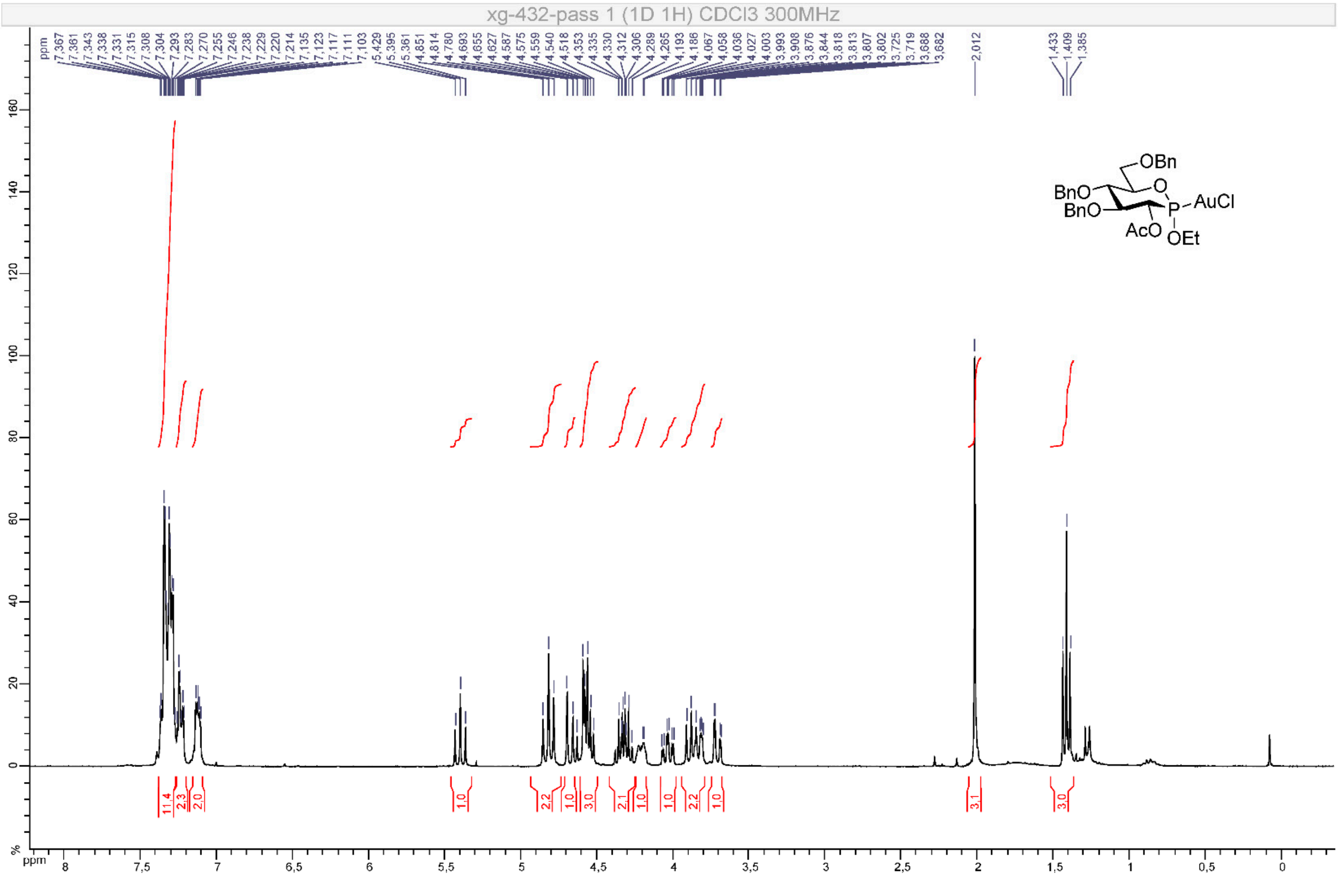

Figure S1. ^{1}H-NMR (300 MHz, $CDCl_3$) of **6a**.

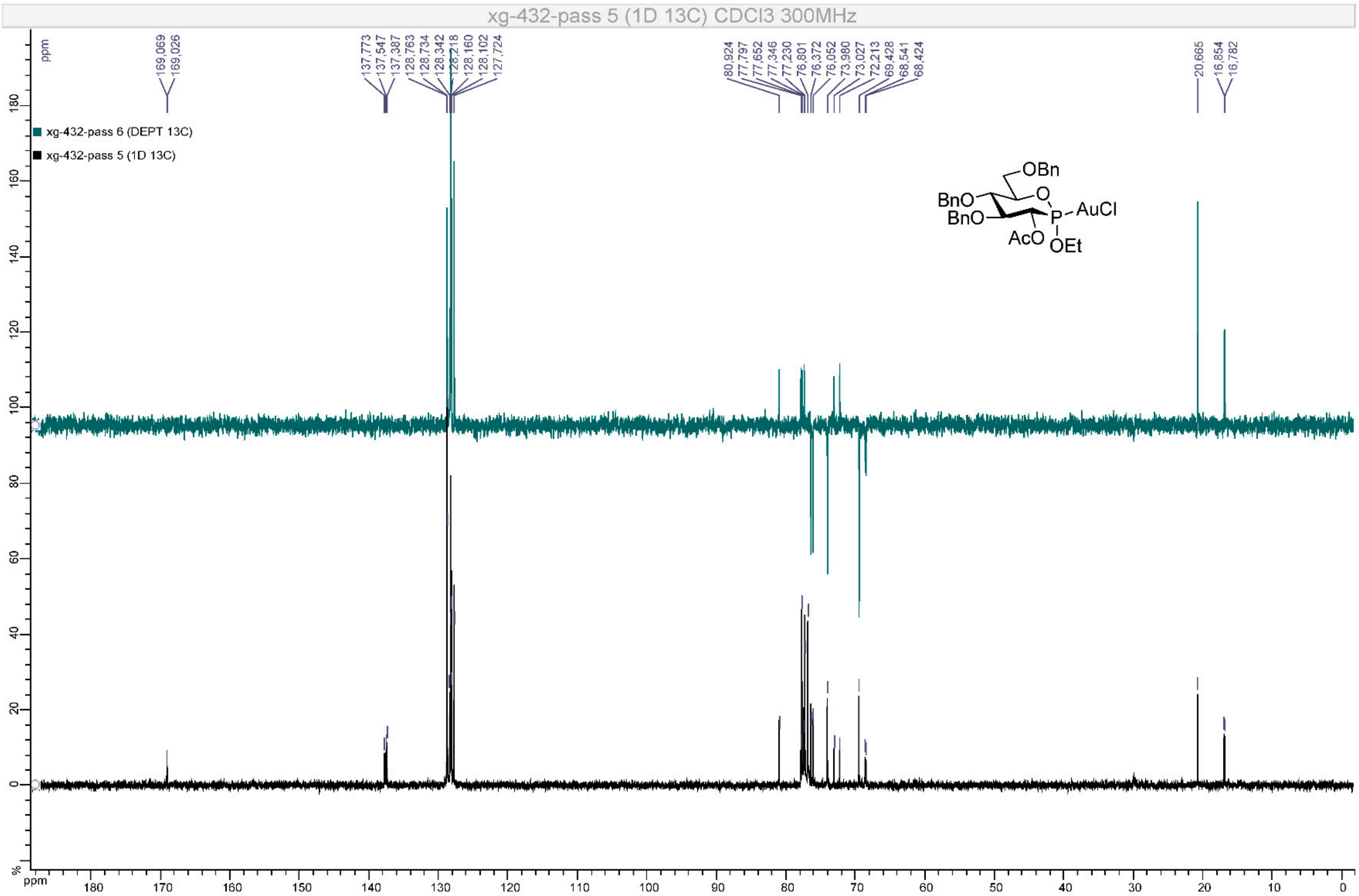

Figure S2. ^{13}C-NMR (75 MHz, $CDCl_3$) **6a**.

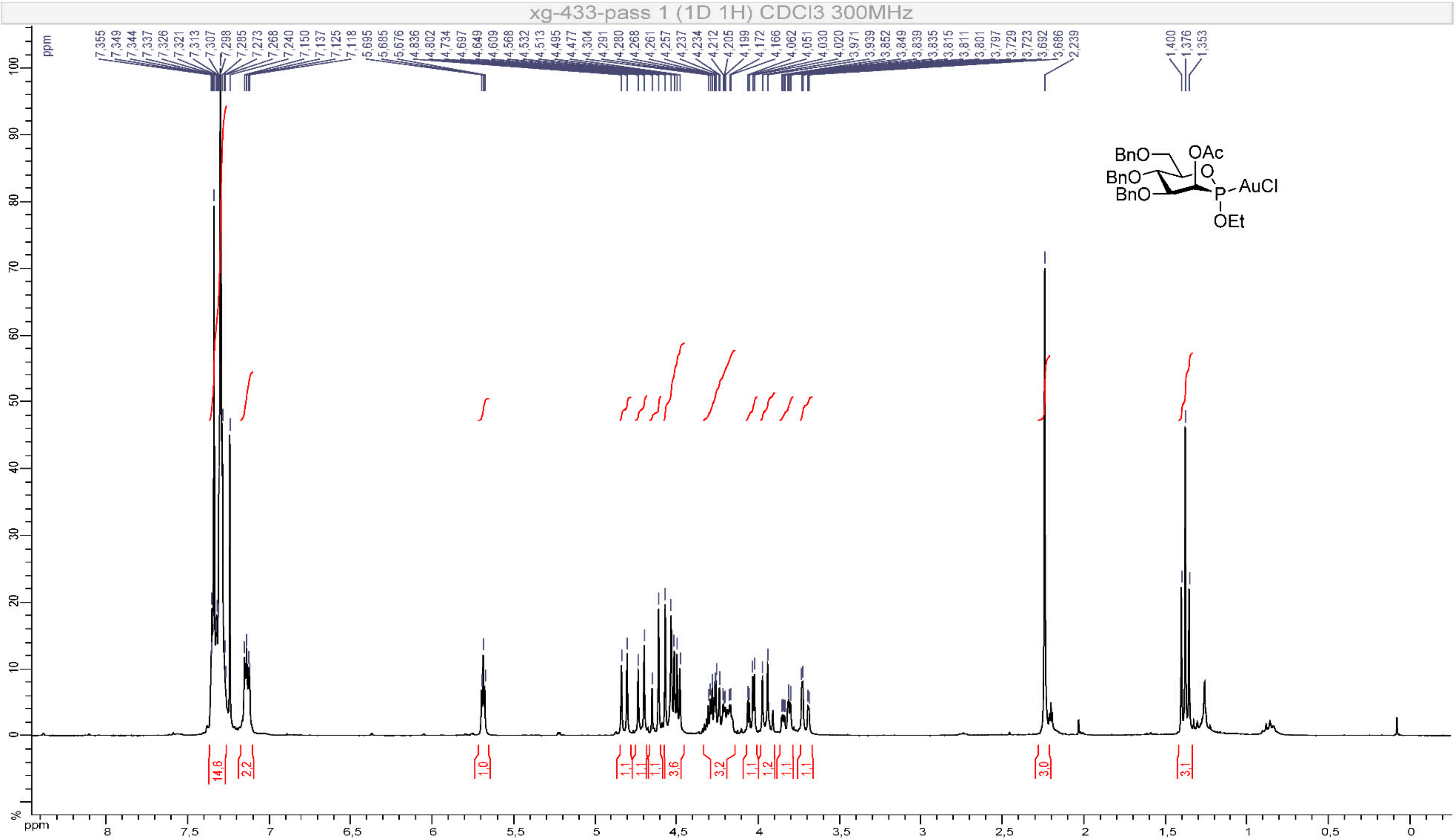

Figure S3. ^{1}H-NMR (300 MHz, $CDCl_3$) **6b**.

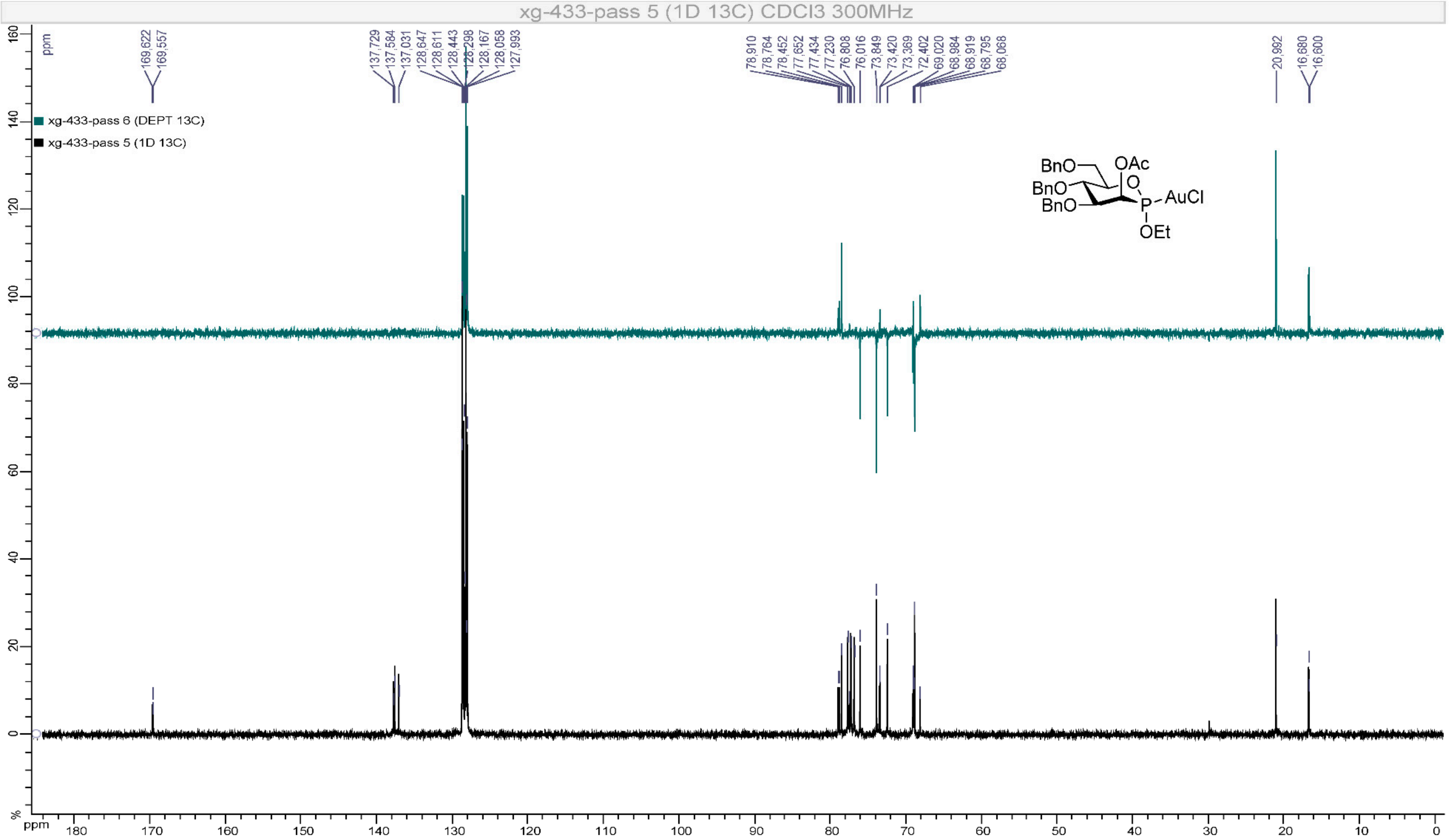

Figure S4. ^{13}C-NMR (75 MHz, $CDCl_3$) **6b**.

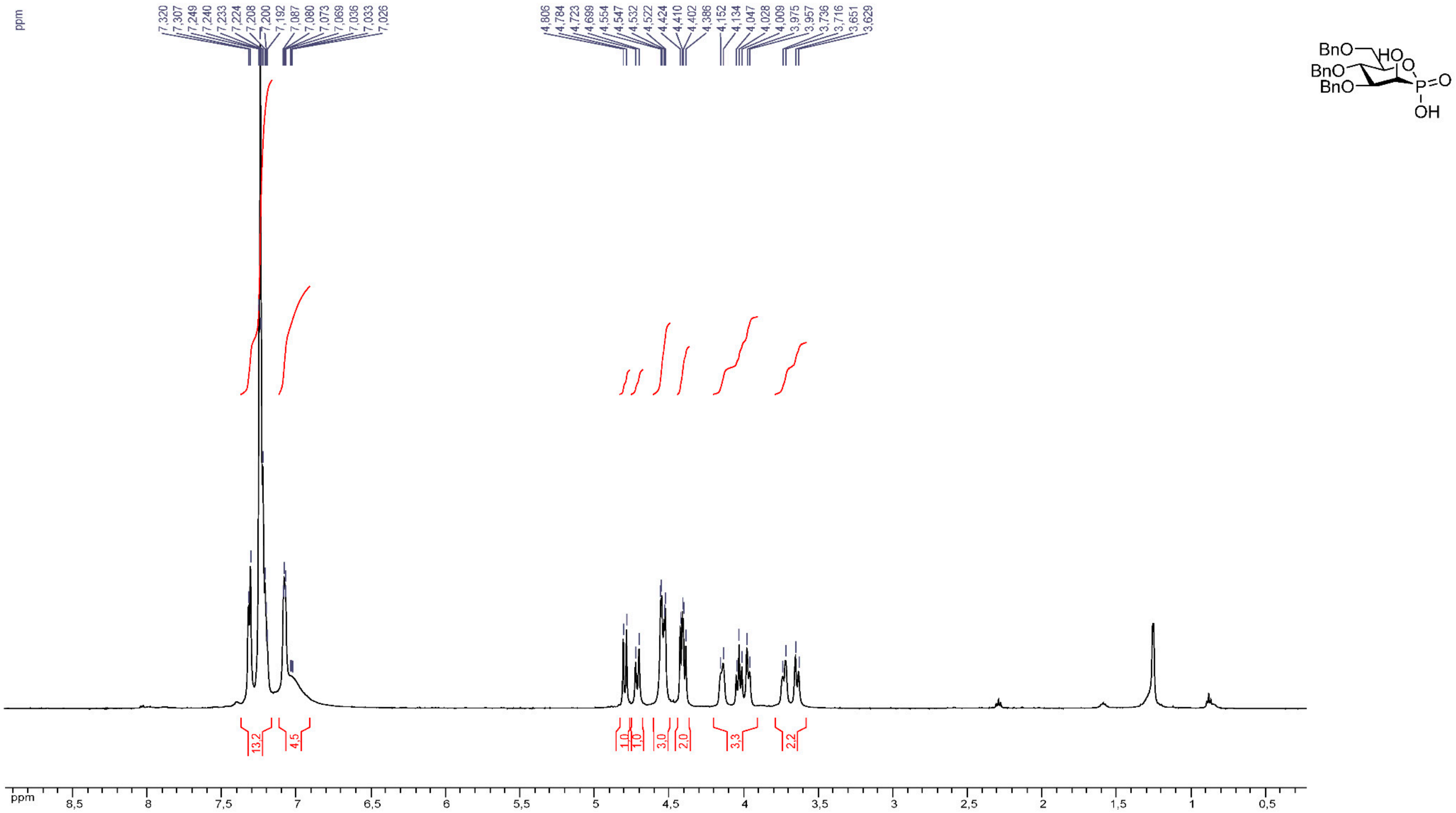

Figure S5. ^{1}H-NMR (300 MHz, $CDCl_3$) **2a**.

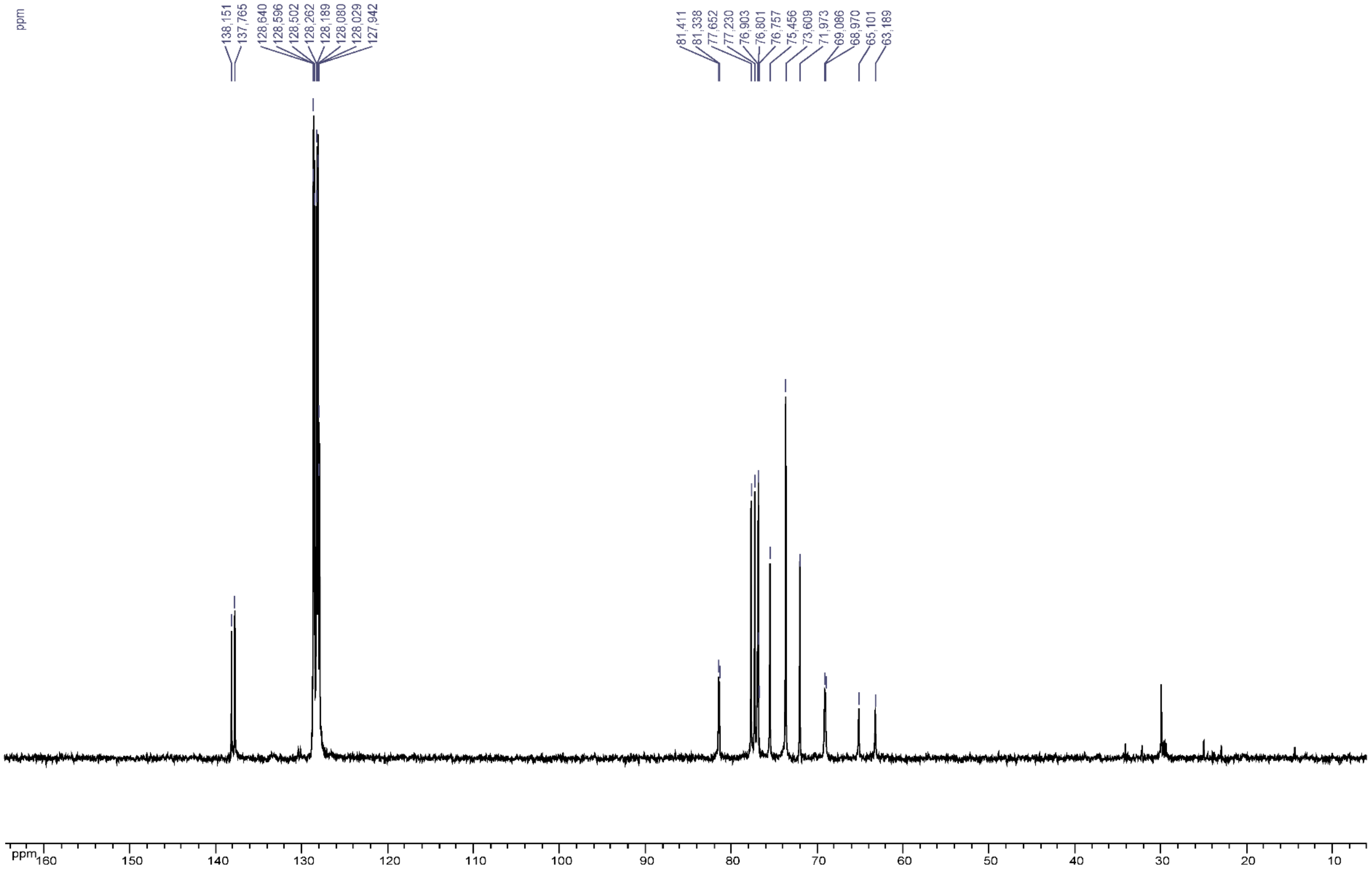

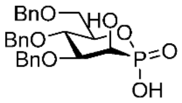

Figure S6. ^{13}C-NMR (75 MHz, $CDCl_3$) **2a**.

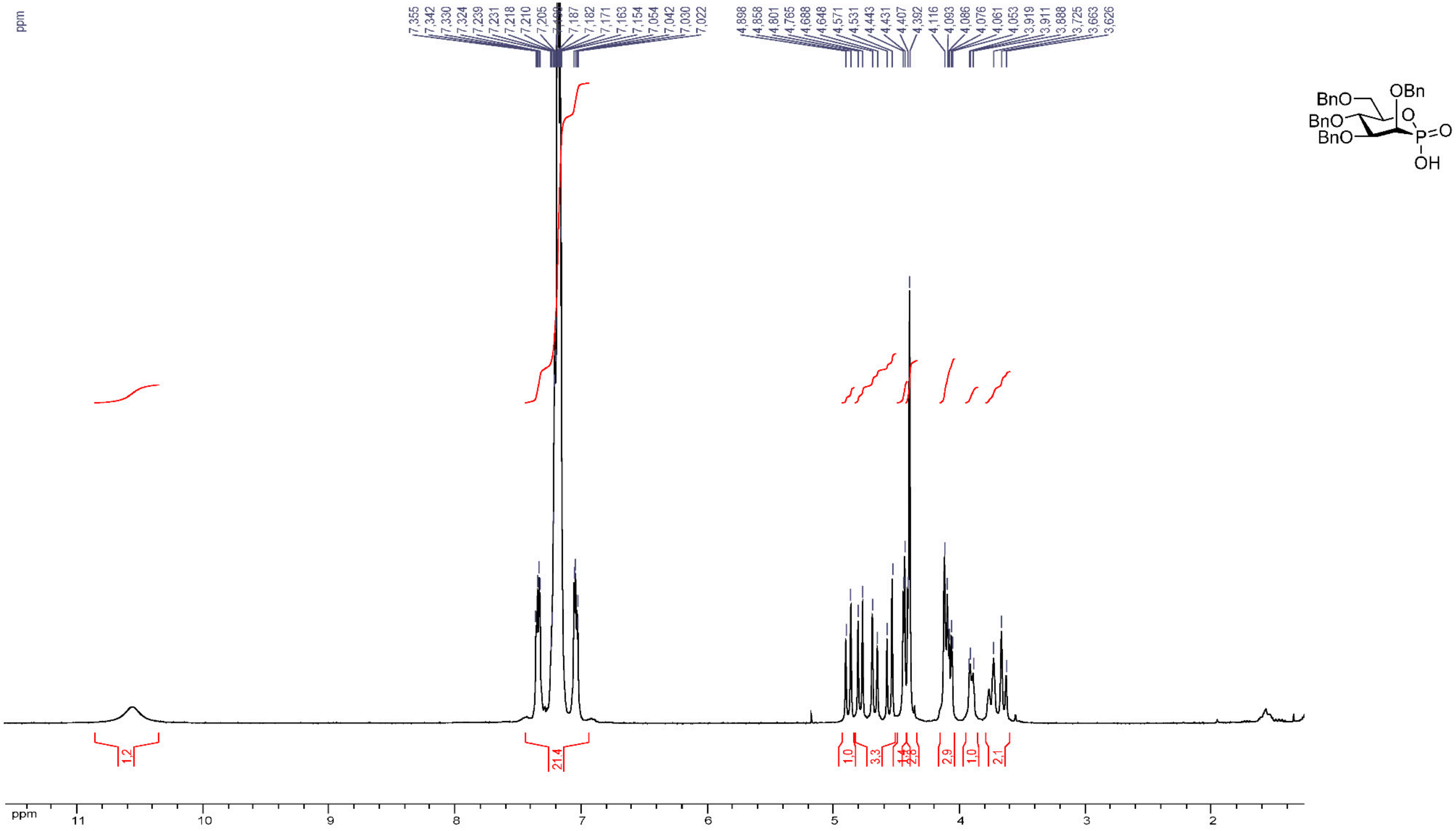

Figure S7. ^{1}H-NMR (500 MHz, $CDCl_3$) **2b**.

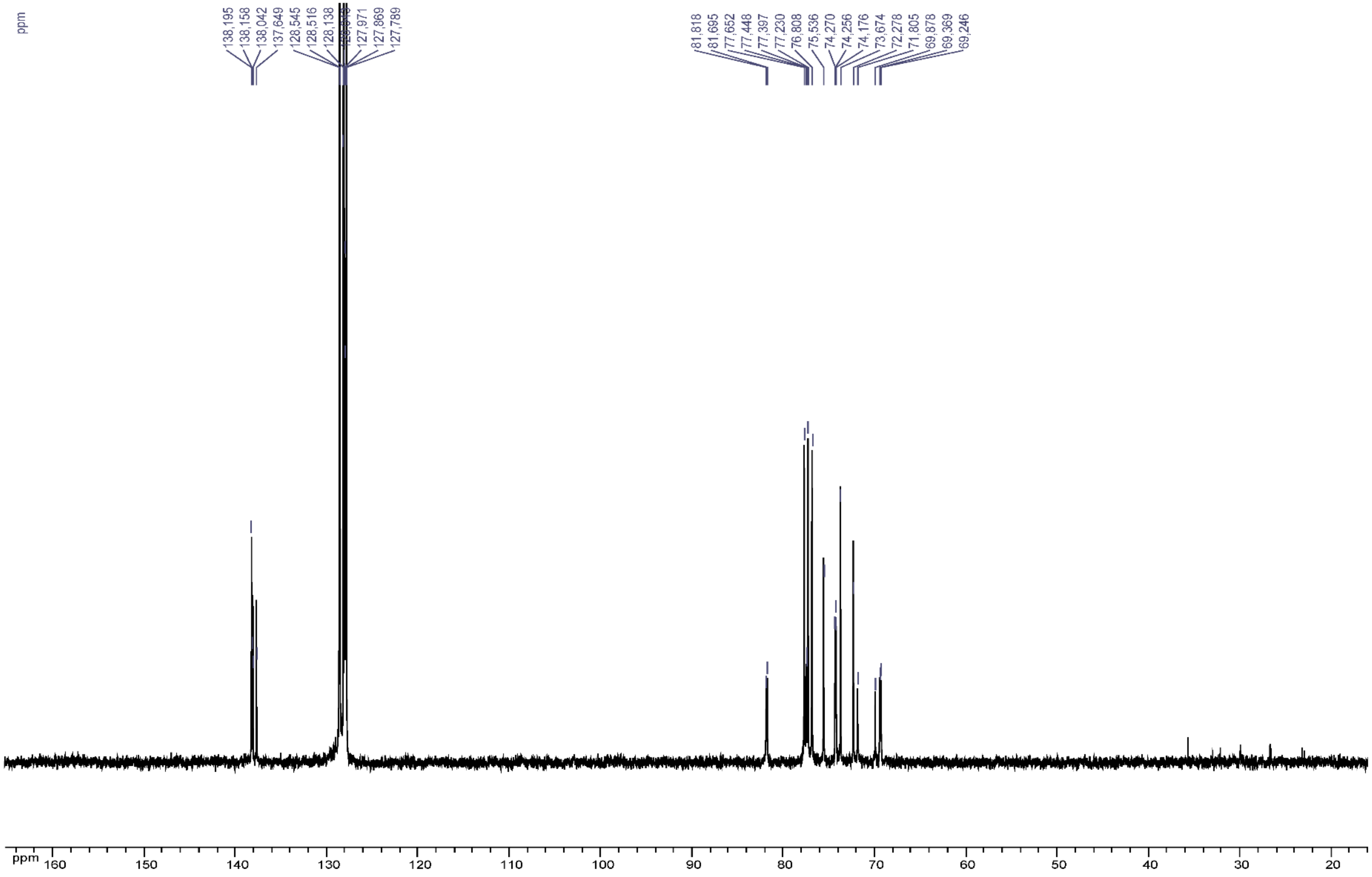

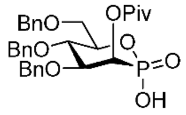

Figure S8. ^{13}C-NMR (75 MHz, $CDCl_3$) **2b**.

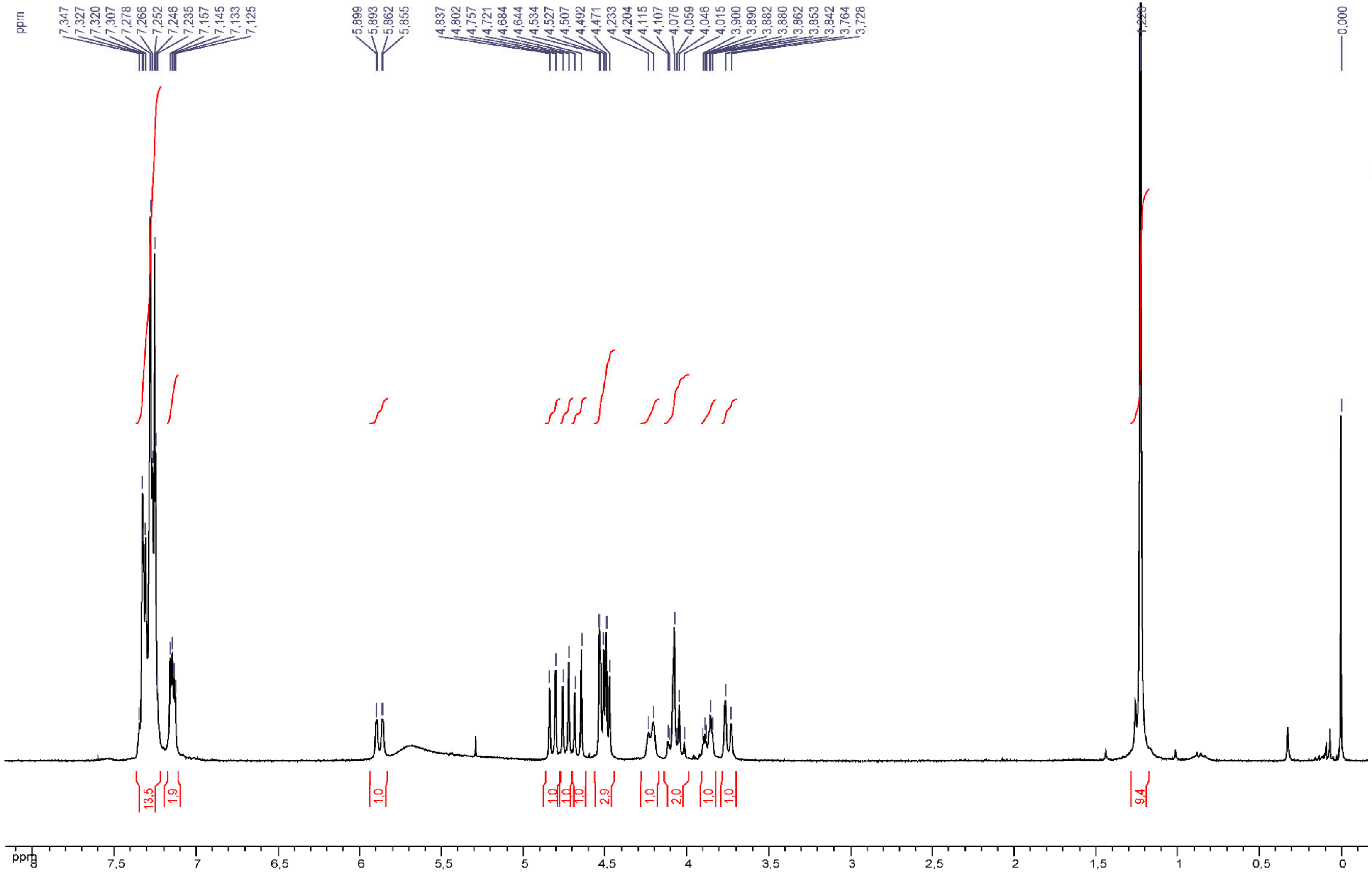

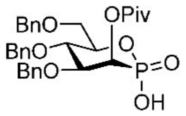

Figure S9. ^{1}H-NMR (500 MHz, $CDCl_3$) **2c**.

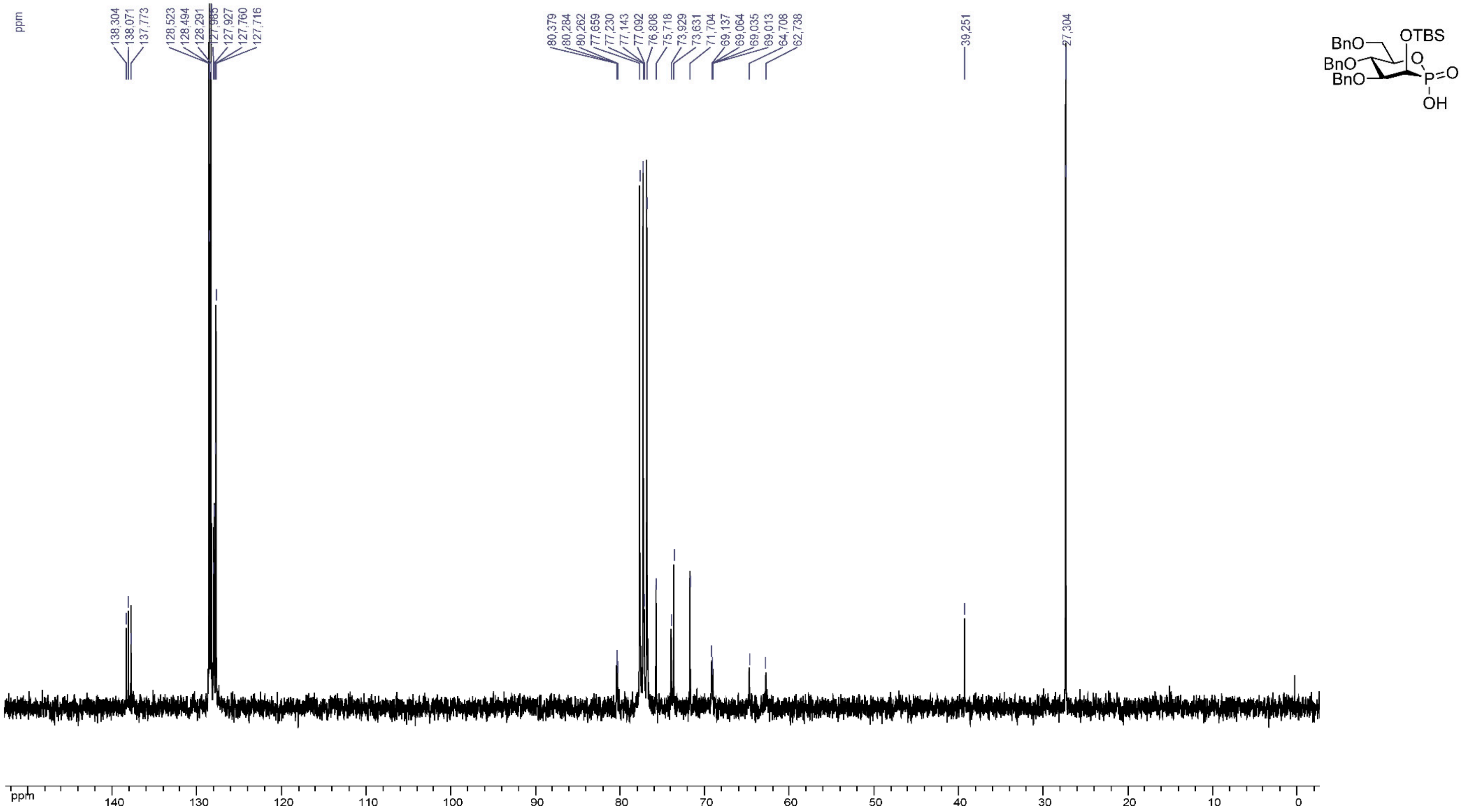

Figure S10. ^{13}C-NMR (75 MHz, $CDCl_3$) **2c**.

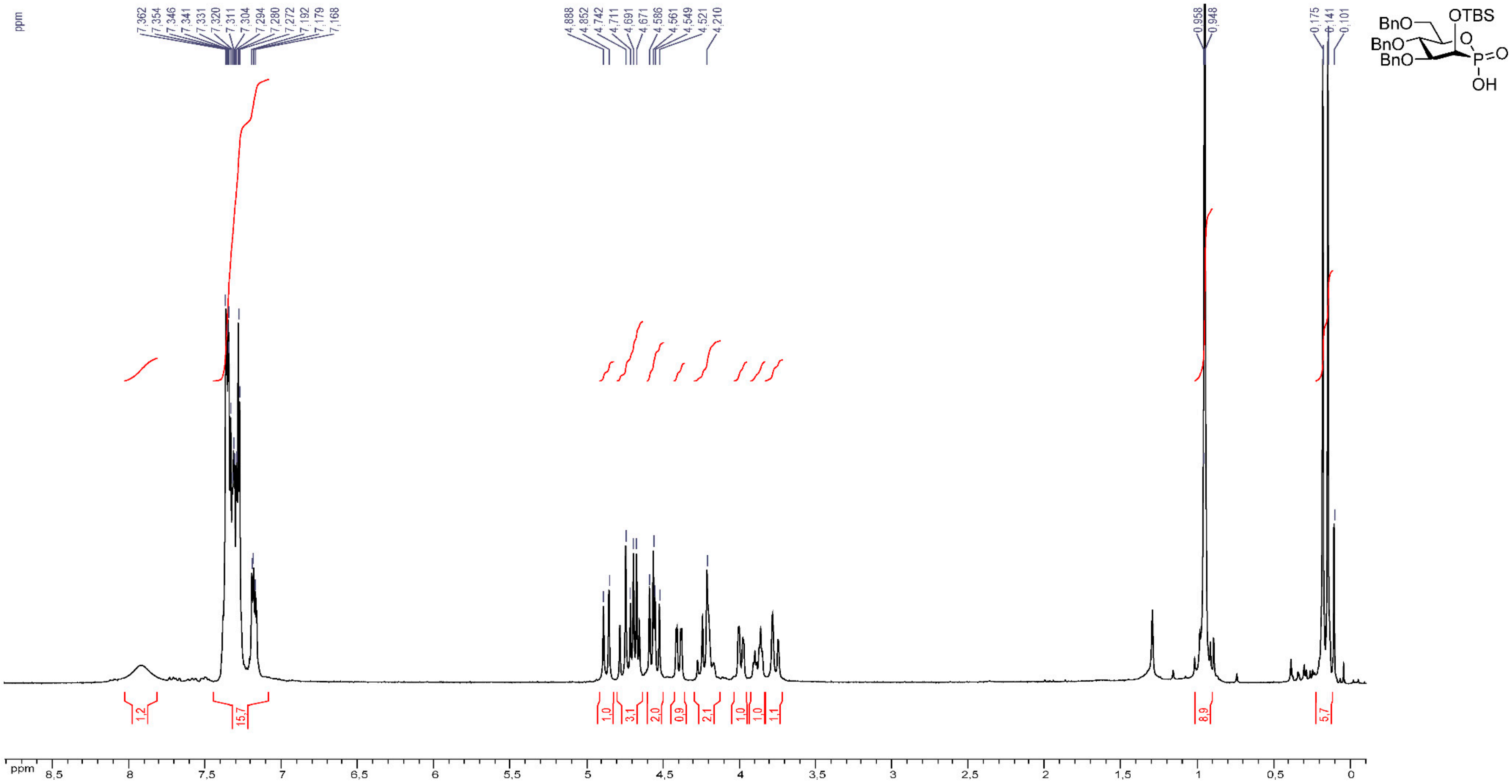

Figure S11. ^{1}H-NMR (300 MHz, $CDCl_3$) **2d**.

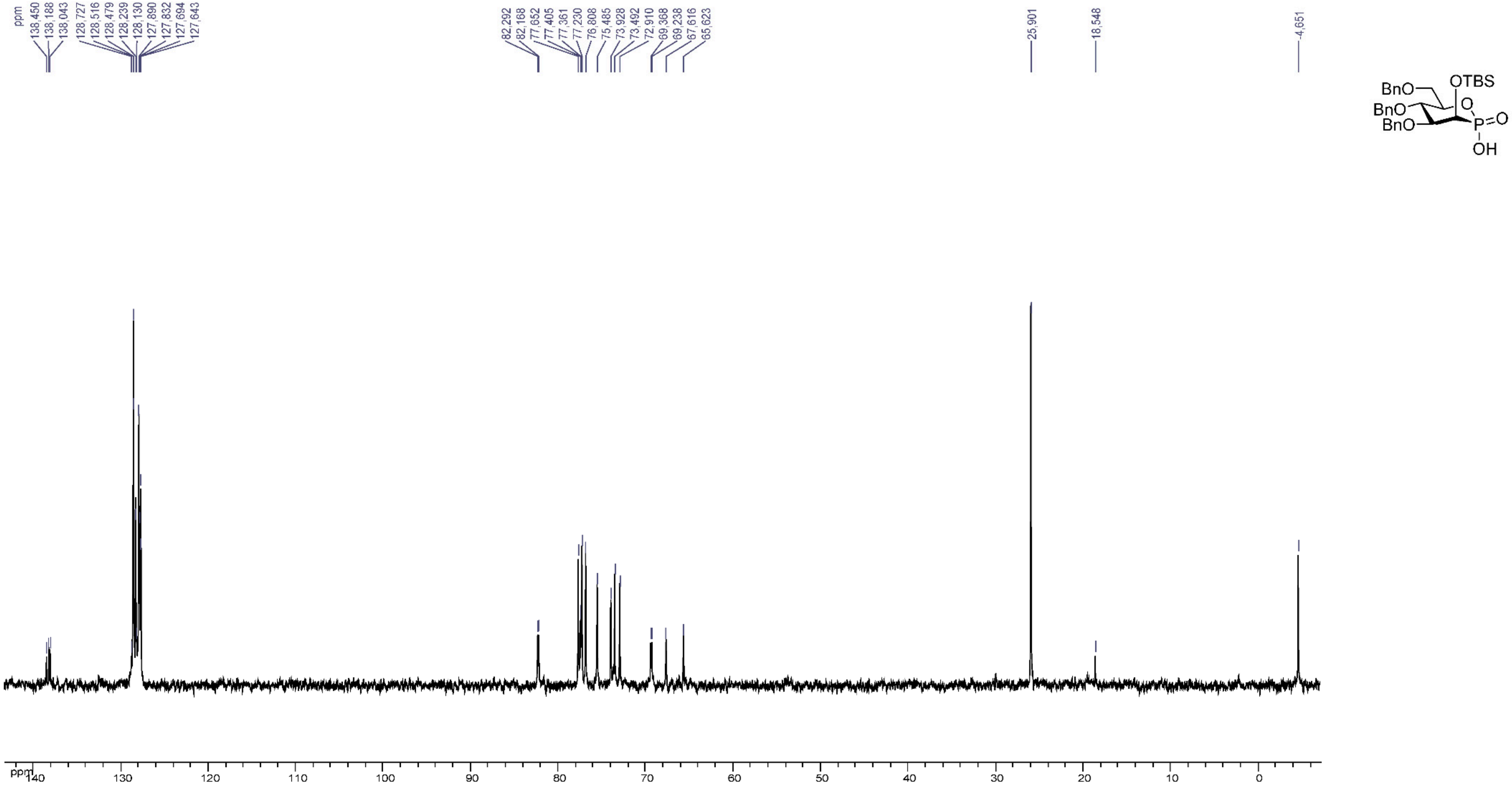

Figure S12. ^{13}C-NMR (75 MHz, $CDCl_3$) **2d**.

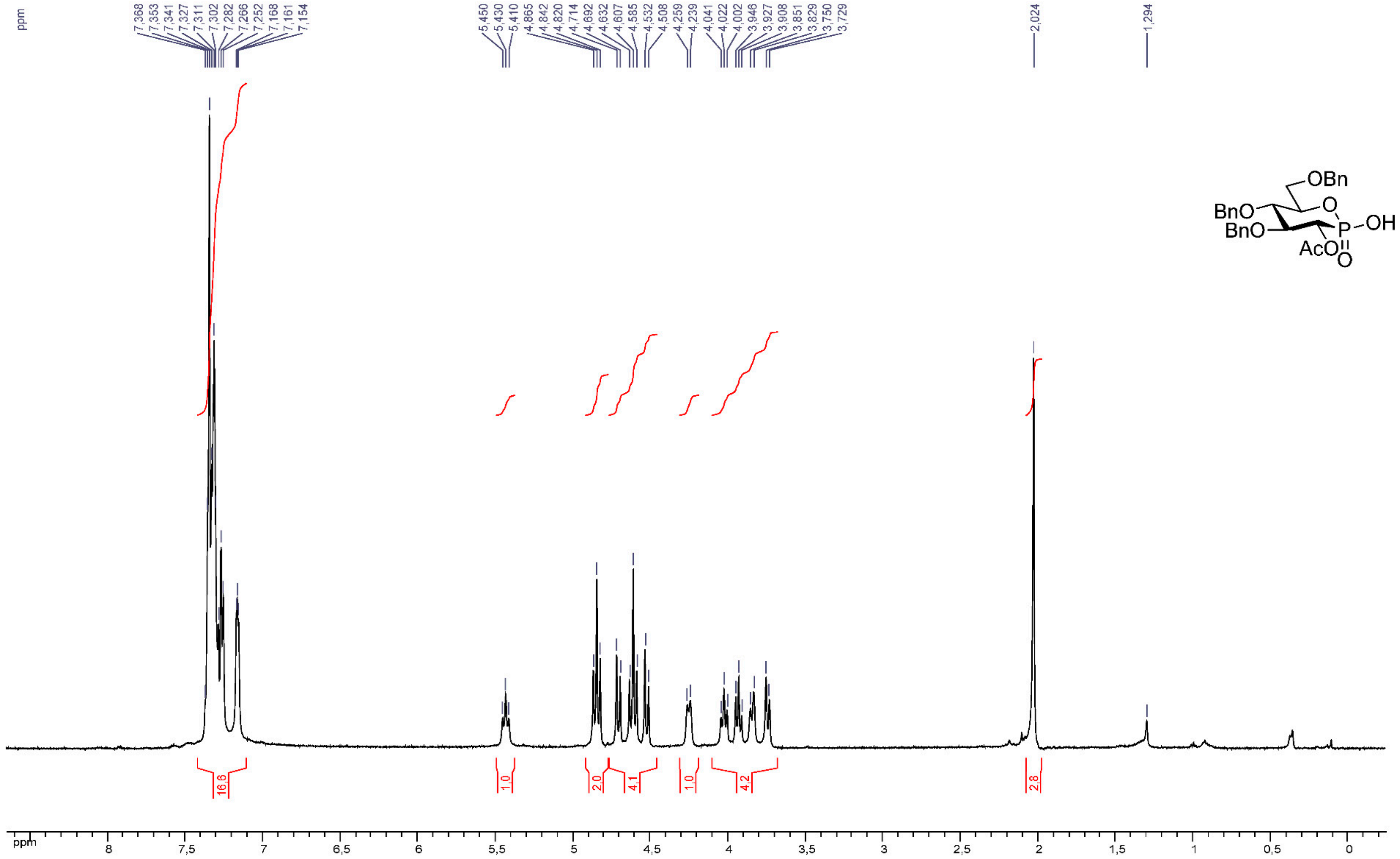

Figure S13. ^{1}H-NMR (500 MHz, $CDCl_3$) **2e**.

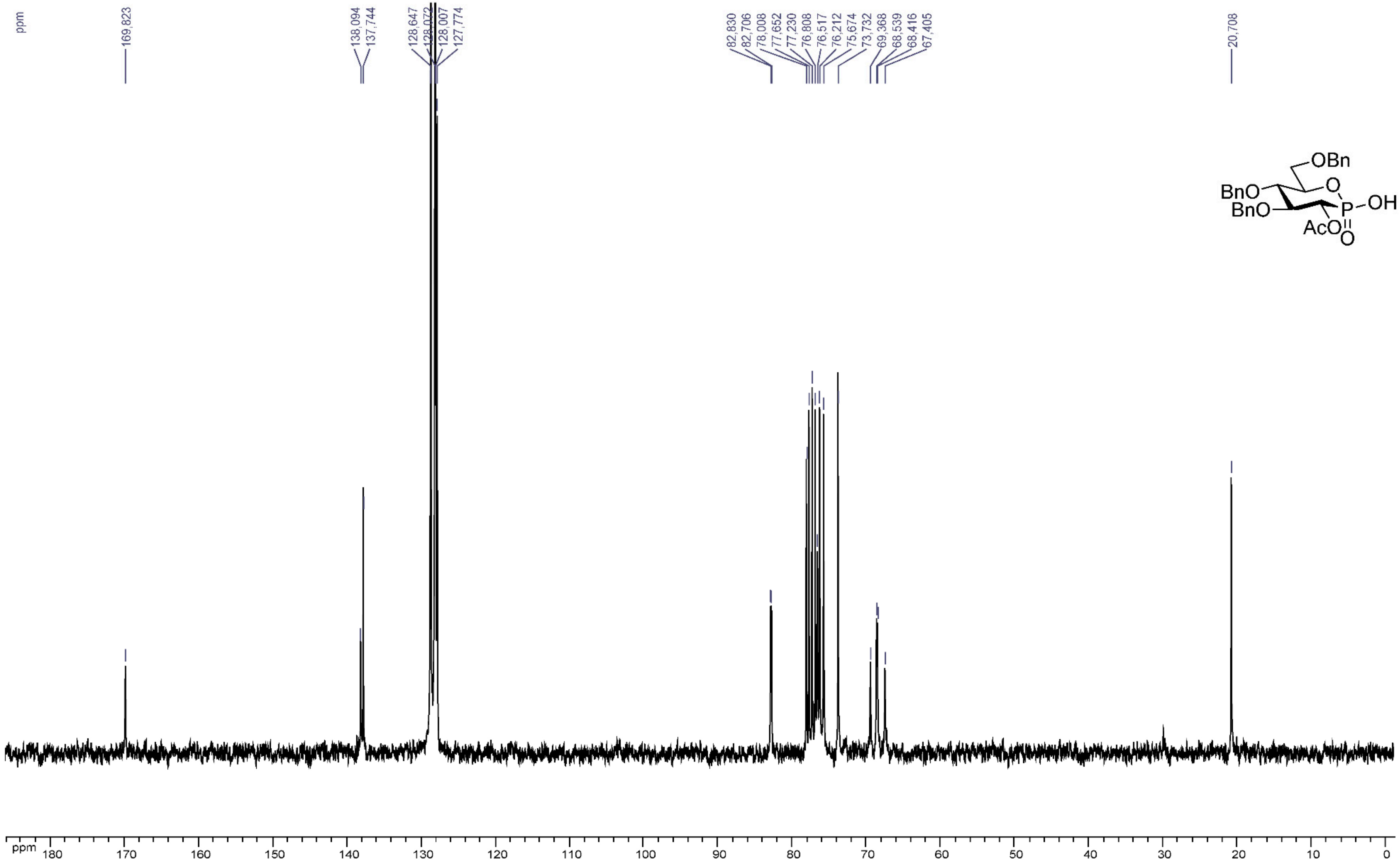

Figure S14. ^{13}C-NMR (75 MHz, MeOD) **2e**.

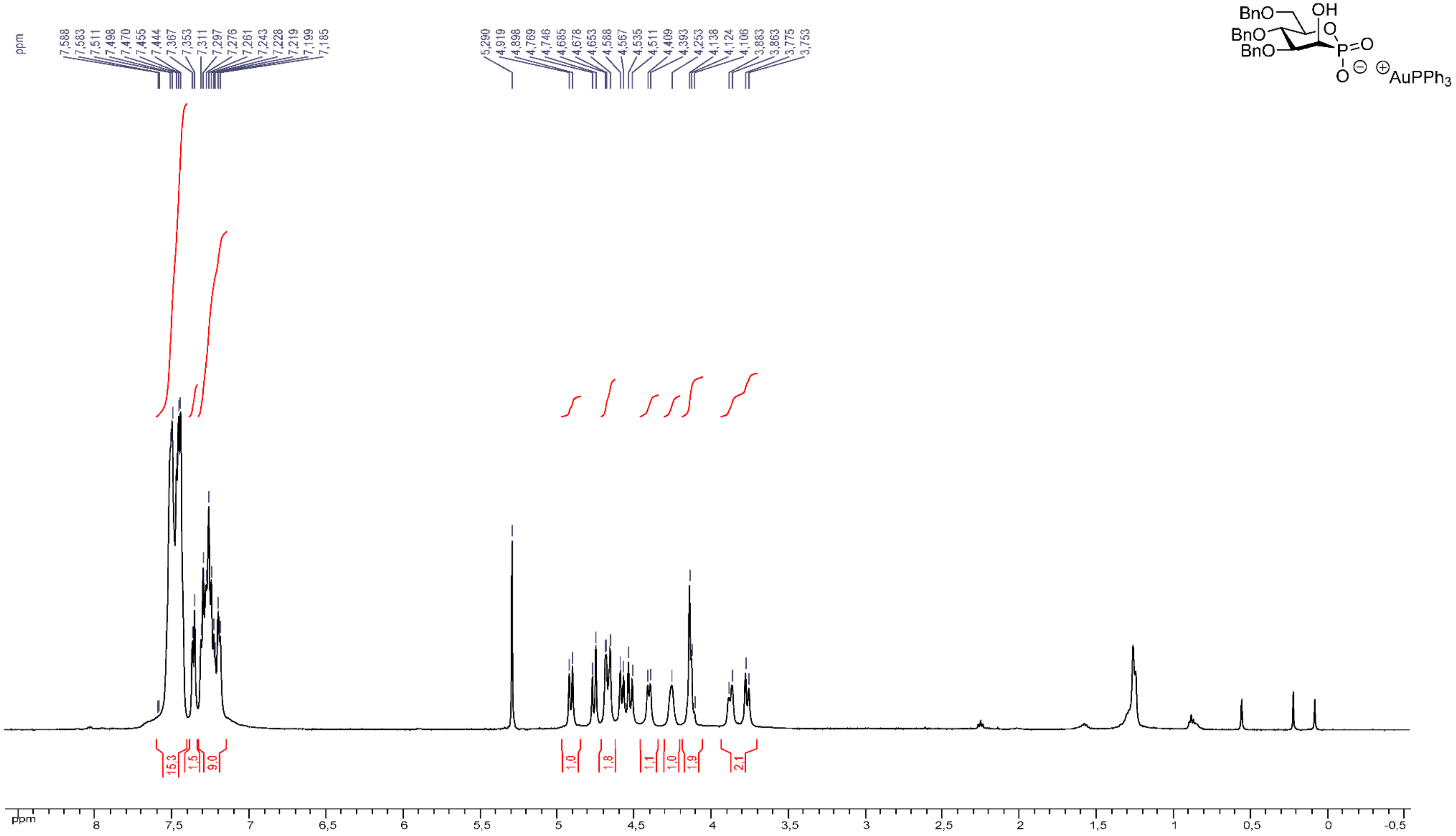

Figure S15. ^{1}H-NMR (500 MHz, $CDCl_3$) **9a**.

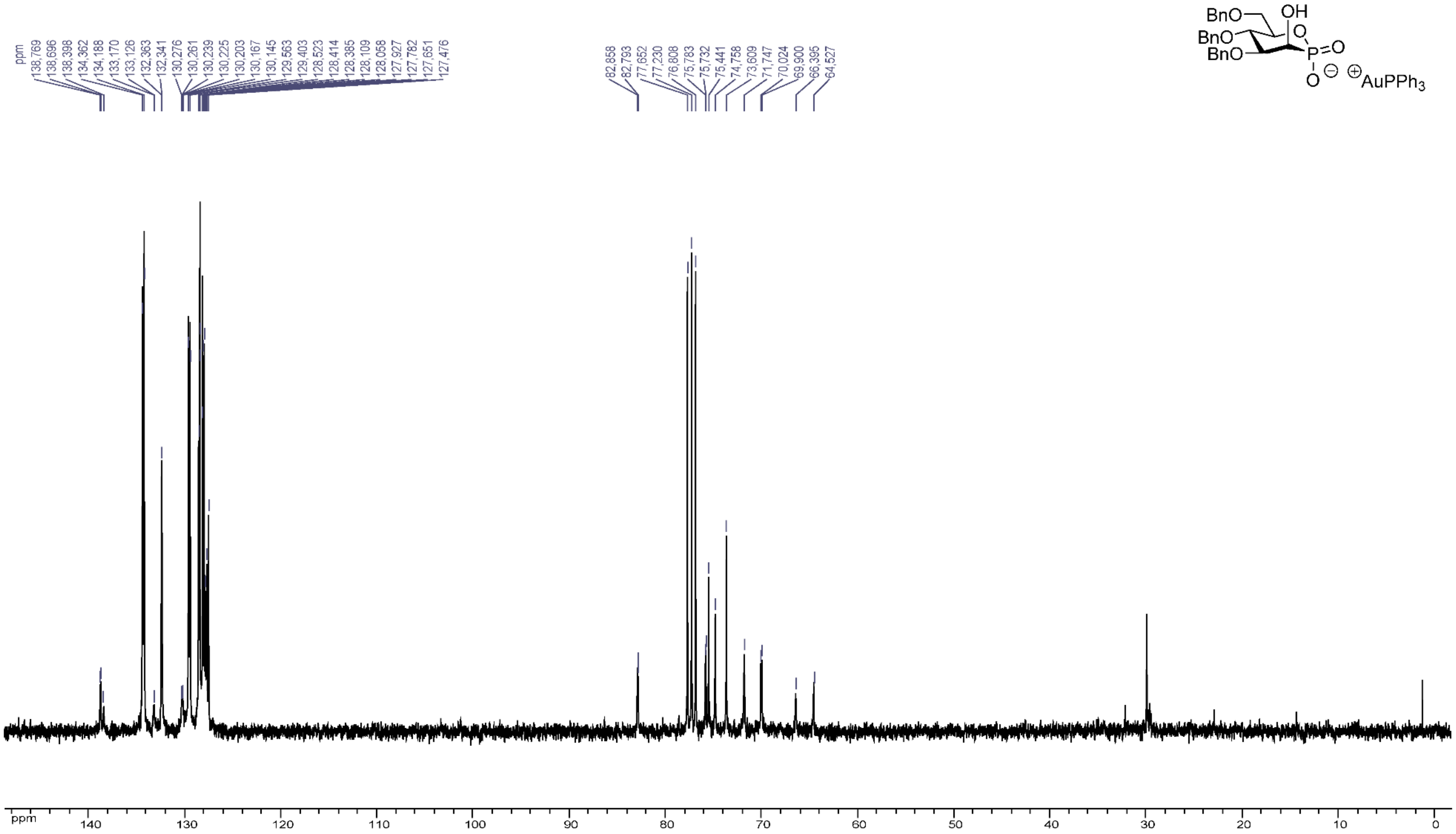

Figure S16. ^{13}C-NMR (75 MHz, $CDCl_3$) **9a**.

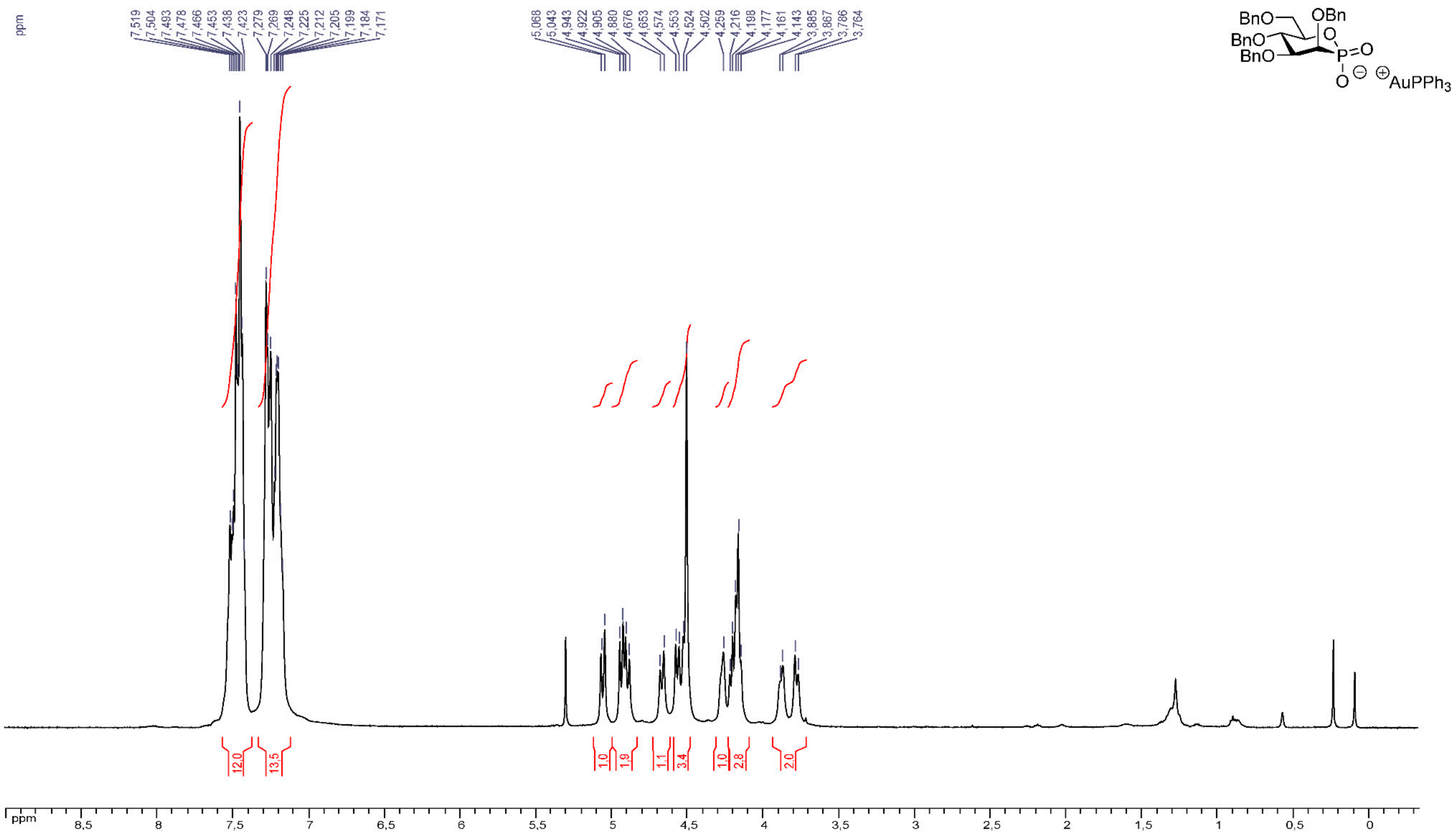

Figure S17. ^{1}H-NMR (500 MHz, $CDCl_3$) **9b**.

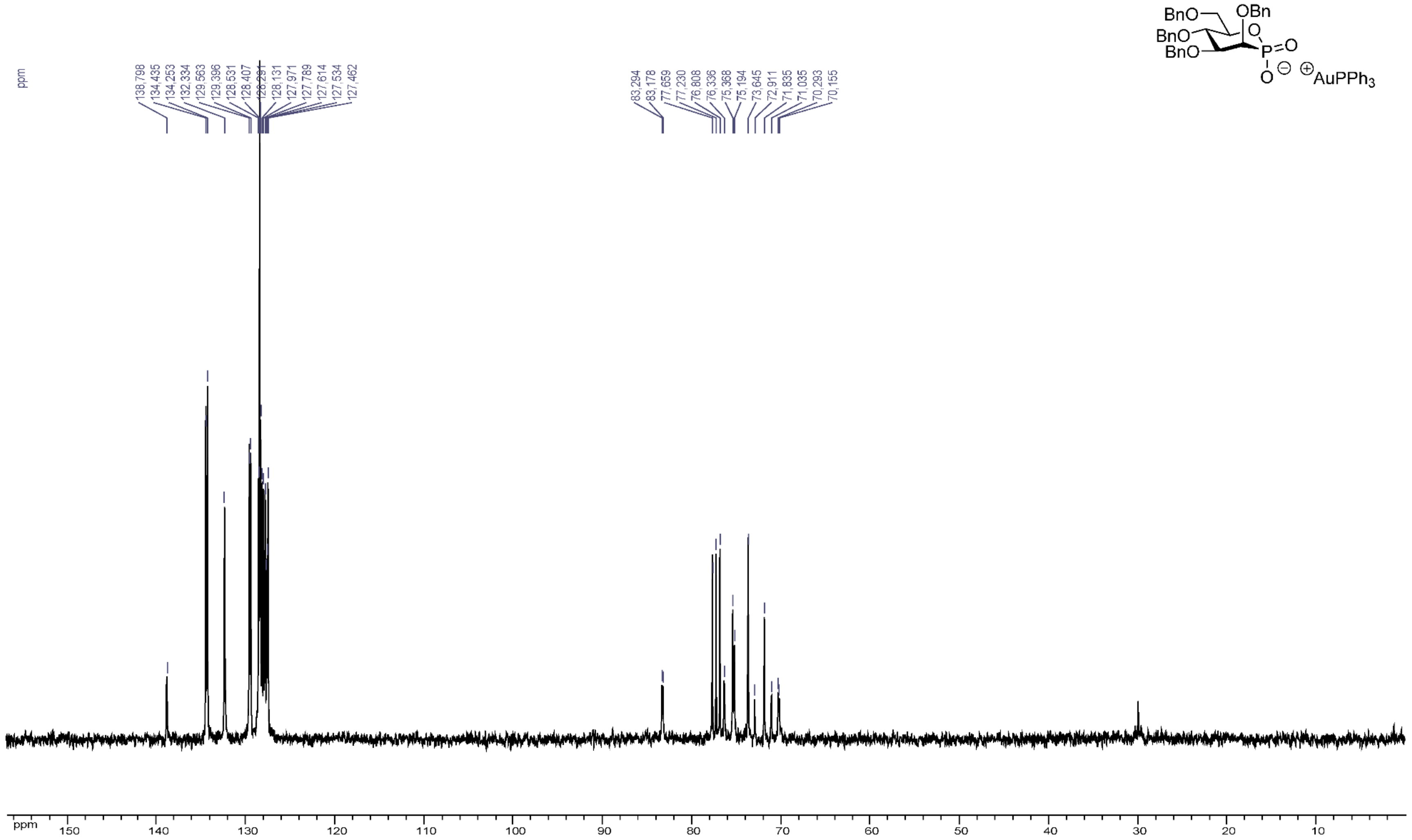

Figure S18. ^{13}C-NMR (75 MHz, $CDCl_3$) **9b**.

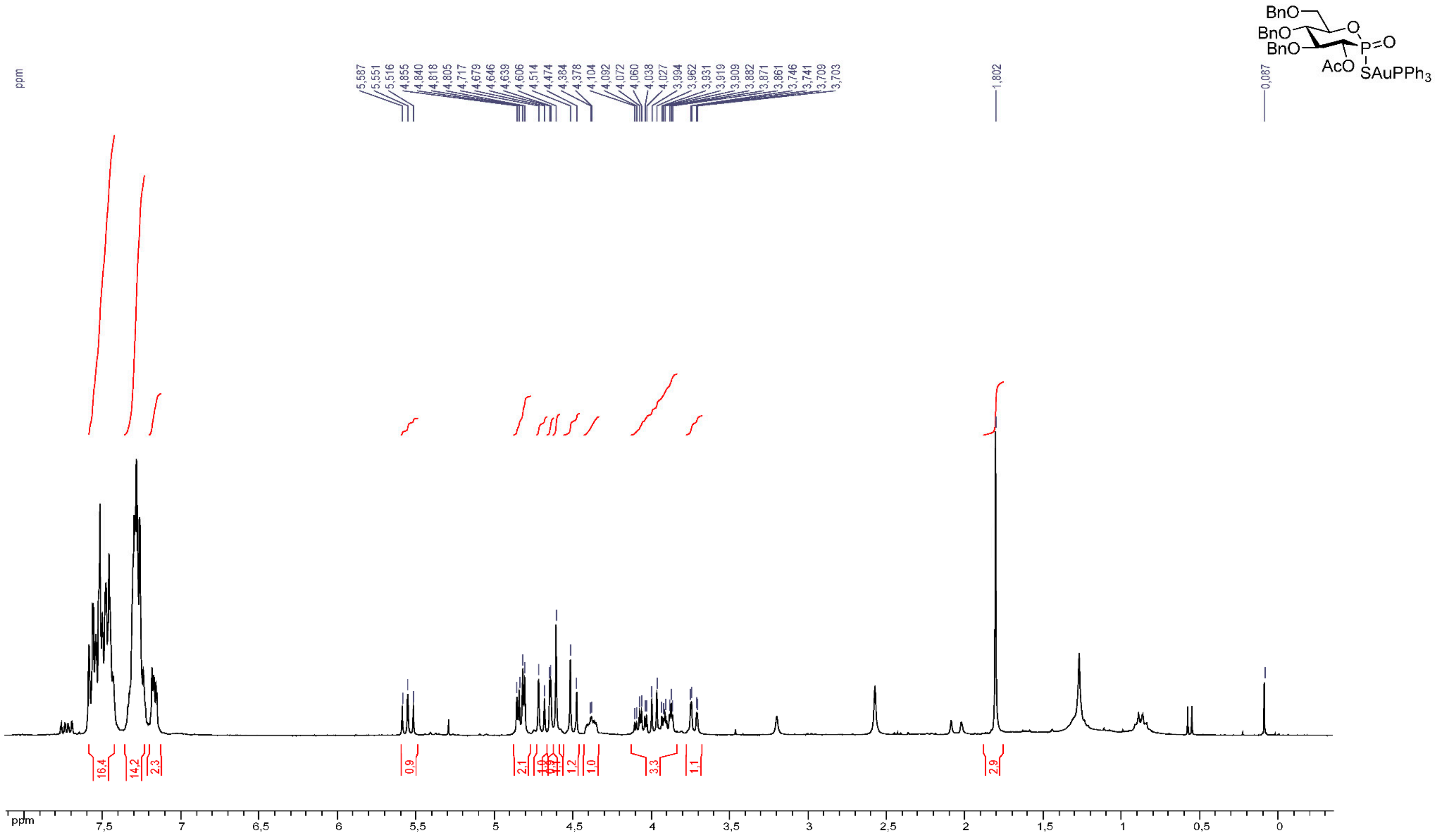

Figure S19. ^{1}H-NMR (300 MHz, $CDCl_3$) **10a**.

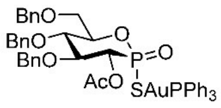

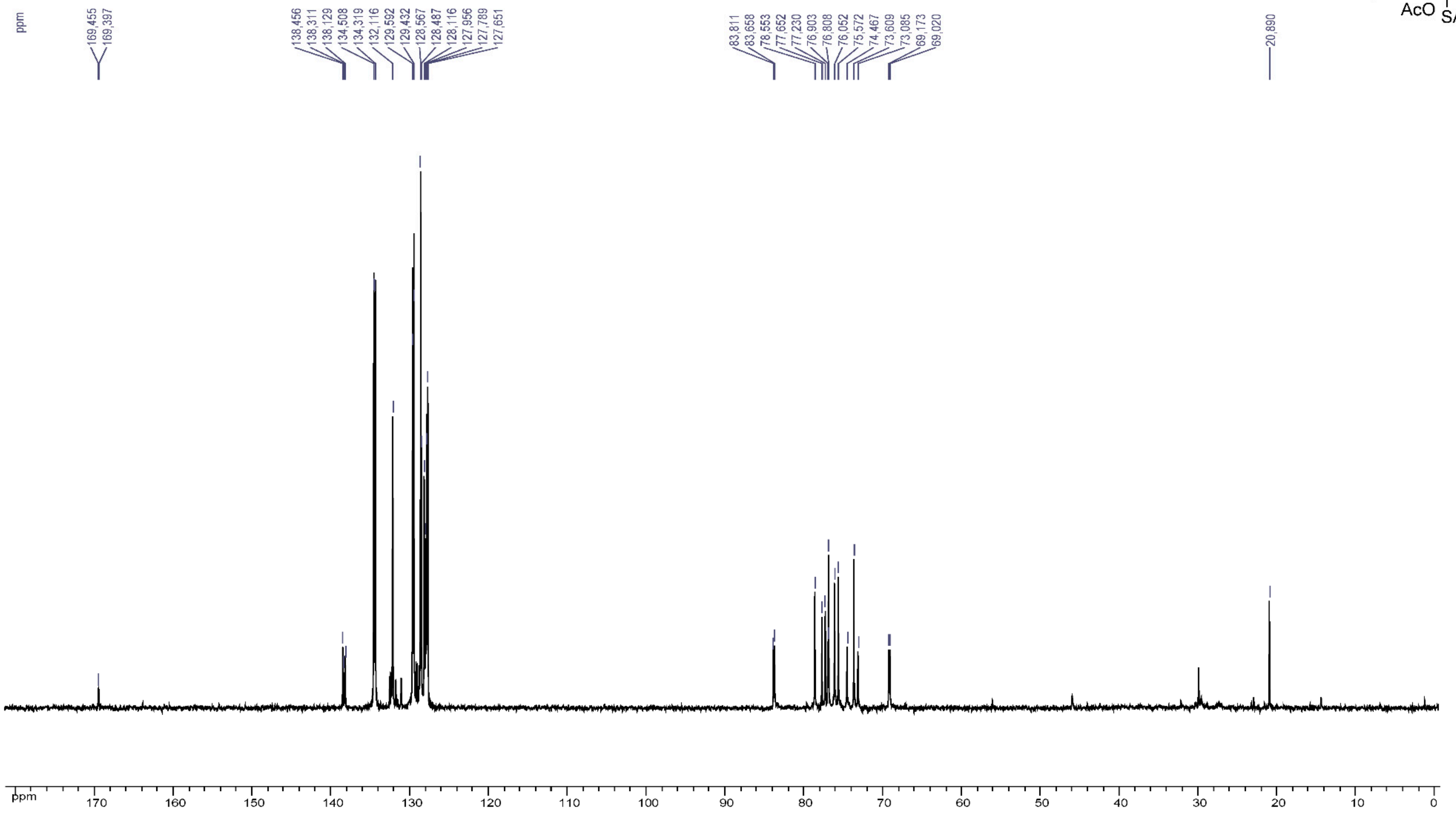

Figure S20. ^{13}C-NMR (75 MHz, $CDCl_3$) **10a**.
